# Supplementary material for: Unravelling single-cell DNA replication timing dynamics using machine learning reveals heterogeneity in cancer progression
Source: Nat Commun. 2025 Feb 8;16:1472. doi: 10.1038/s41467-025-56783-0 (PMC11807193; doi:10.1038/s41467-025-56783-0)
Supplement: Supplementary file 1 — Supplementary Information [file 41467_2025_56783_MOESM1_ESM.pdf]

Supplementary Information

Unravelling single-cell DNA replication timing dynamics using machine learning reveals heterogeneity in cancer progression

Joseph M. Josephides, Chun-Long Chen

Supplementary Figures
Supplementary Tables

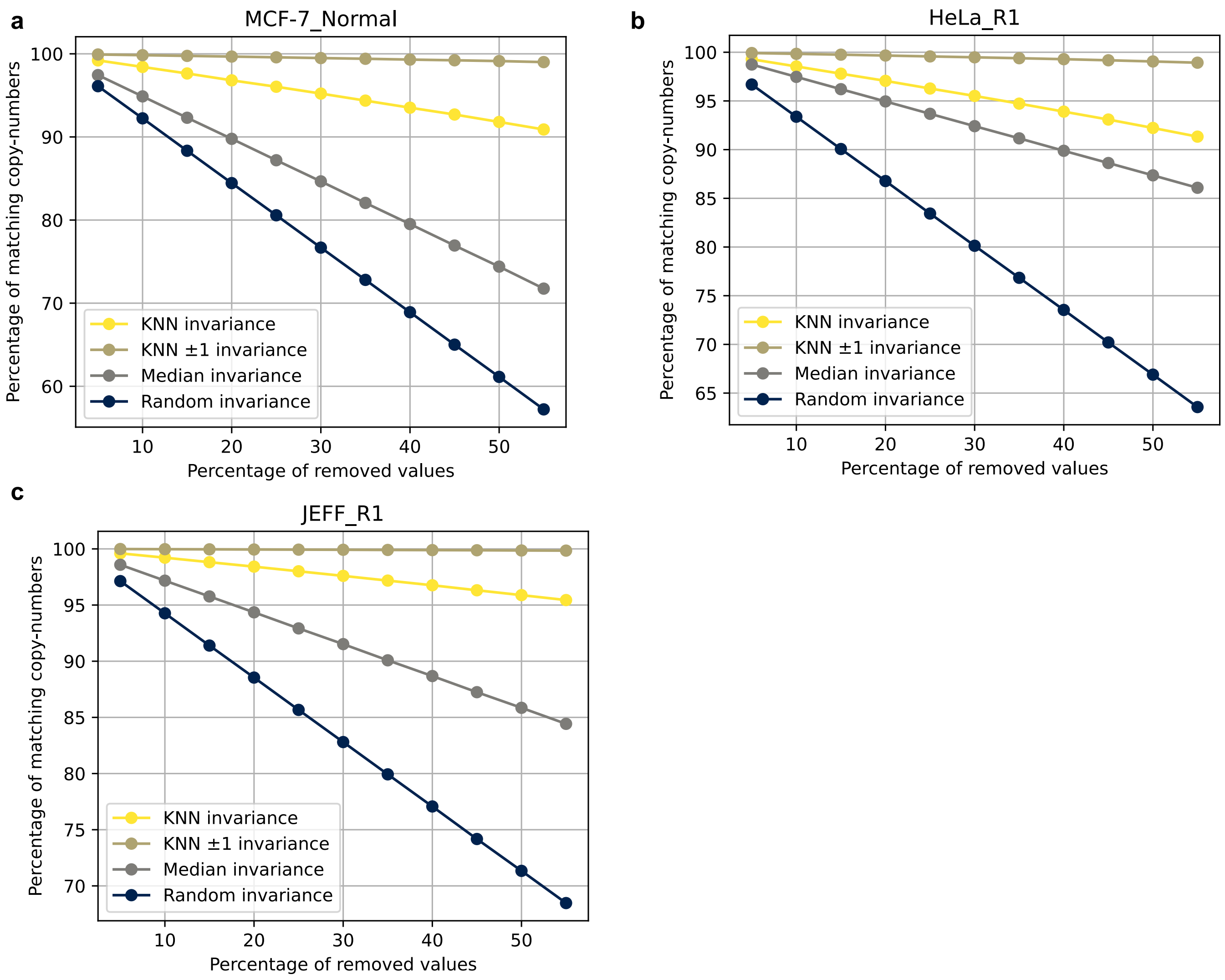

Supplementary Figure S1. KNN imputation is an efficient method compensating for single-cell copy-number scarcity. Copy-numbers from (a) MCF-7 (normal replicate; n=2,321 cells), (b) HeLa (replicate 1; n=459 cells), and (c) JEFF (replicate 1; n=952 cells) were used to simulate missing values (data from ref.[37]) in 100 kb windows which underwent random value removal ranging from 5 to 55% of the total number of values in the single-cell copy-number matrix (regions/cells). KNN, median and random imputations were performed while KNN imputed values that varied by ±1 copy-number were calculated. These four metrics were compared to the original values for copy-number matrix-wide invariance. Four simulations with different seeds were performed to obtain the mean and standard deviation (SD) values for each percentage of removed values with the KNN accuracy. However, the SD values (ranging from 0.024 to 0.0819; average 0.0456 for MCF-7 for example) were too small to be visible on the plot. Source data are provided as a Source Data file.

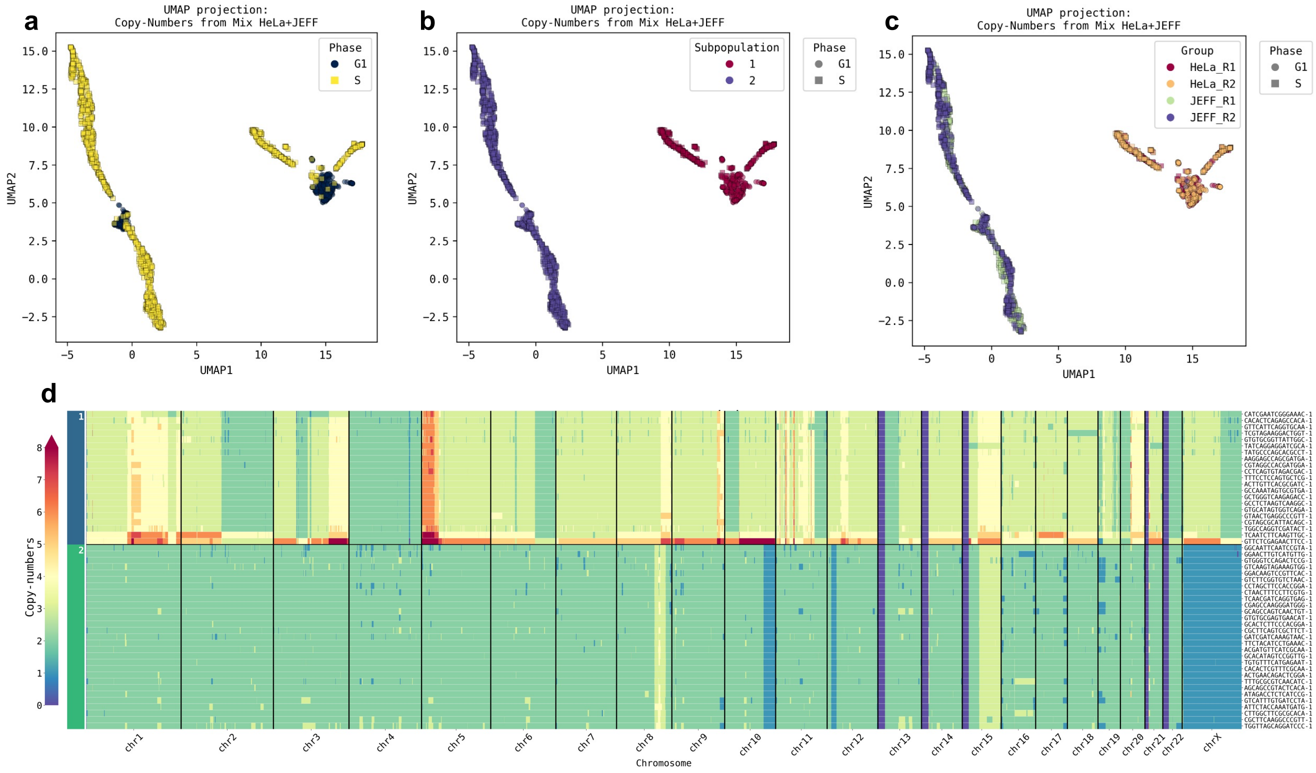

Supplementary Figure S2. scCNV distinctions with unsupervised learning. a-c. UMAP pane of JEFF (n=1,455 cells) and HeLa (n=752 cells) samples coloured by replication state (a), subpopulation (b) and replicate state of final detected groups (c). (d) Randomly selected cells (n=50 cells) and their genome-wide single-cell copy-numbers. Source data are provided as a Source Data file.

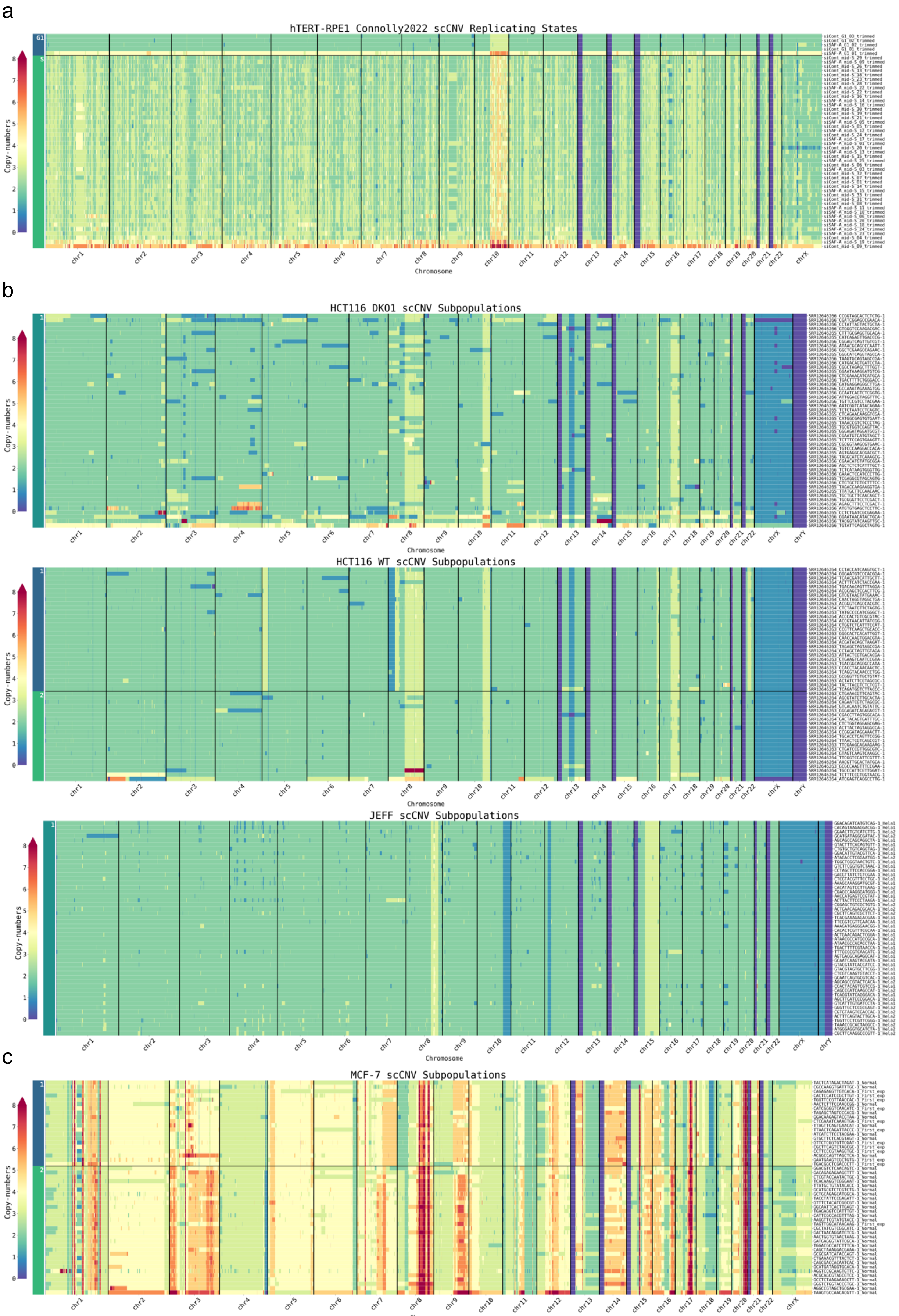
a
hTERT-RPE1 Connolly2022 scCNV Replicating States
G1
S
Copy-numbers
Chromosome
b
HCT116 DKO1 scCNV Subpopulations
HCT116 WT scCNV Subpopulations
JEFF scCNV Subpopulations
c
MCF-7 scCNV Subpopulations
chr1
chr2
chr3
chr4
chr5
chr6
chr7
chr8
chr9
chr10
chr11
chr12
chr13
chr14
chr15
chr16
chr17
chr18
chr19
chr20
chr21
chr22
chrX
chrY

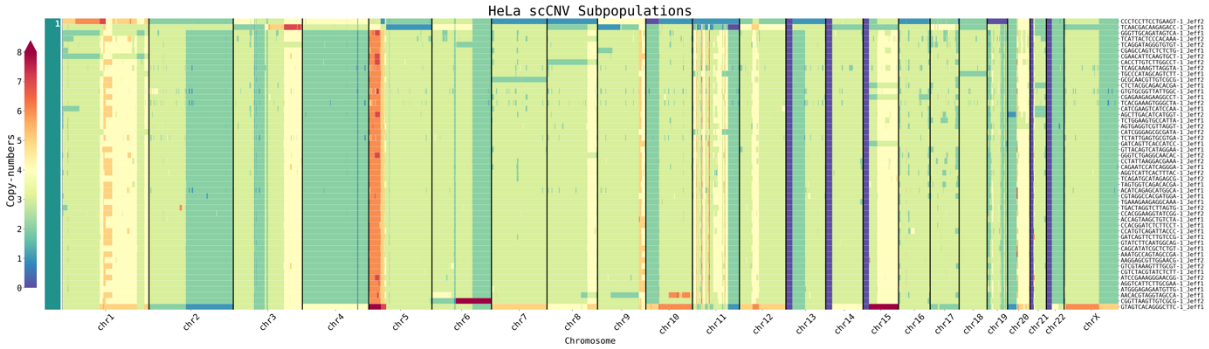
HeLa scCNV Subpopulations
Copy-numbers
Chromosome
chr1 chr2 chr3 chr4 chr5 chr6 chr7 chr8 chr9 chr10 chr11 chr12 chr13 chr14 chr15 chr16 chr17 chr18 chr19 chr20 chr21 chr22 chrX

d

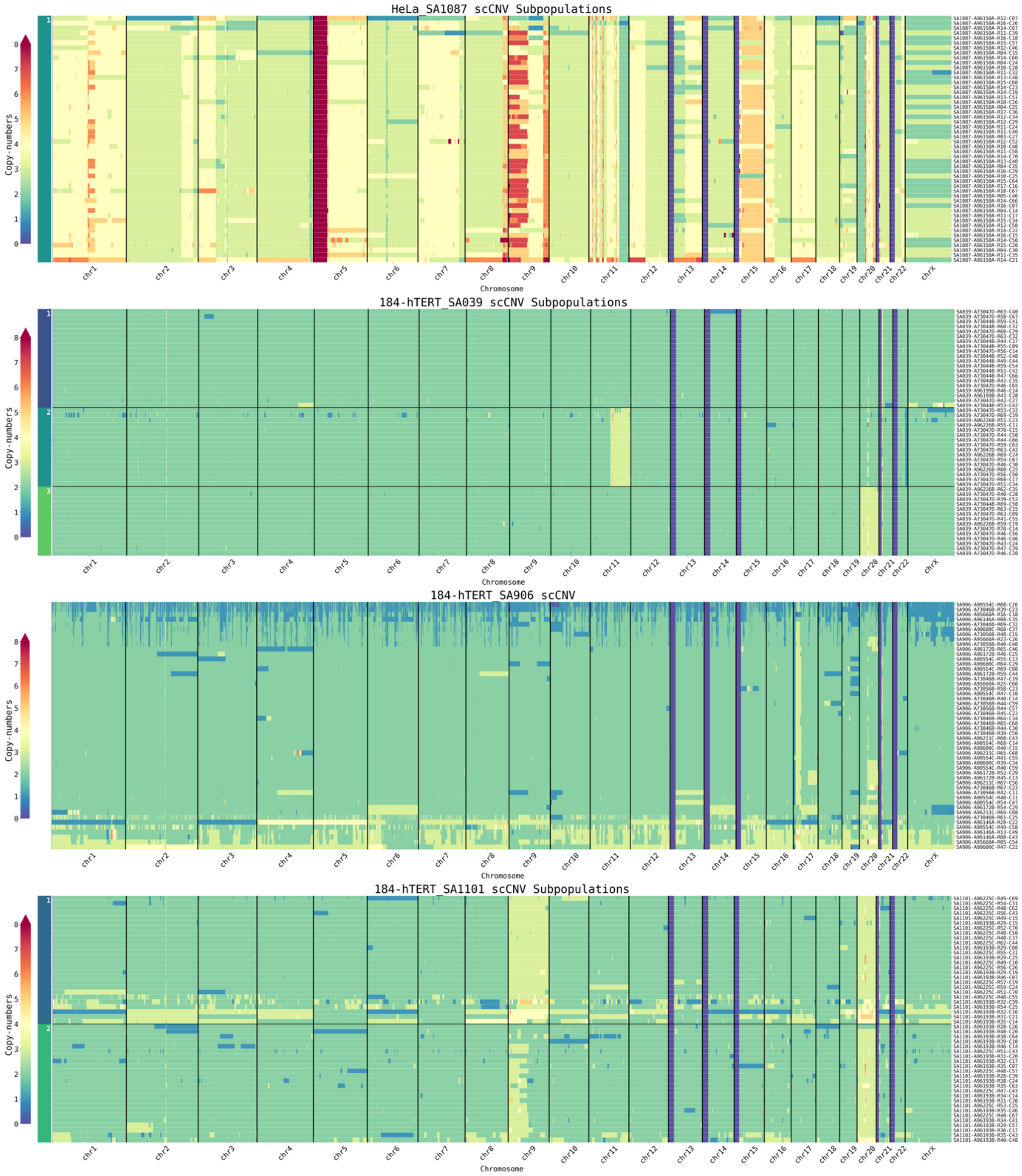
HeLa_SA1087 scCNV Subpopulations
184-hTERT_SA039 scCNV Subpopulations
184-hTERT_SA906 scCNV
184-hTERT_SA1101 scCNV Subpopulations
Copy-numbers
Chromosome
chr1 chr2 chr3 chr4 chr5 chr6 chr7 chr8 chr9 chr10 chr11 chr12 chr13 chr14 chr15 chr16 chr17 chr18 chr19 chr20 chr21 chr22 chrX

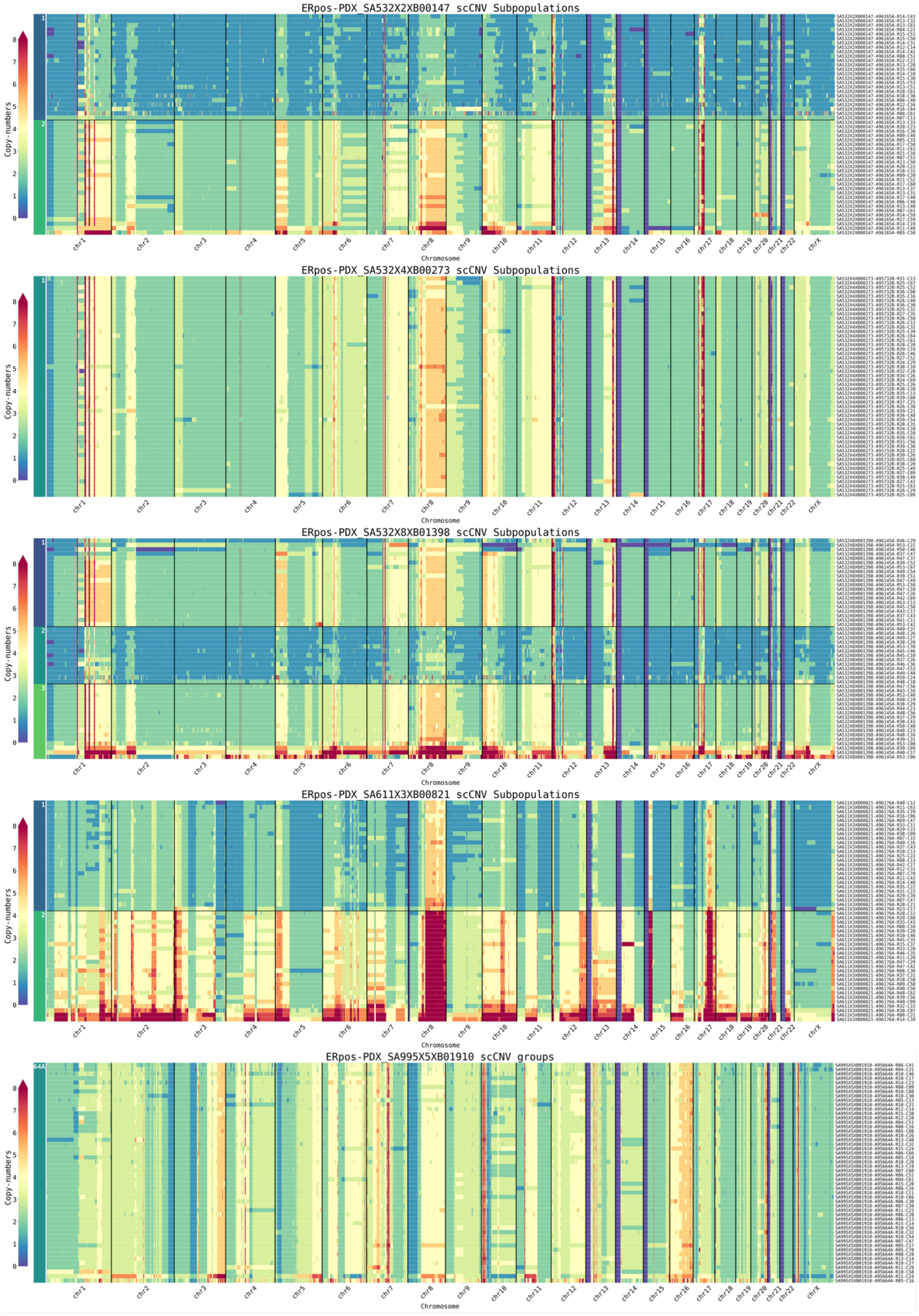
ERpos-PDX_SA532X2XB00147 scCNV Subpopulations
ERpos-PDX_SA532X4XB00273 scCNV Subpopulations
ERpos-PDX_SA532X8XB01398 scCNV Subpopulations
ERpos-PDX_SA611X3XB00821 scCNV Subpopulations
ERpos-PDX_SA995X5XB01910 scCNV groups
Copy-numbers
Chromosome
chr1
chr2
chr3
chr4
chr5
chr6
chr7
chr8
chr9
chr10
chr11
chr12
chr13
chr14
chr15
chr16
chr17
chr18
chr19
chr20
chr21
chr22
chrX

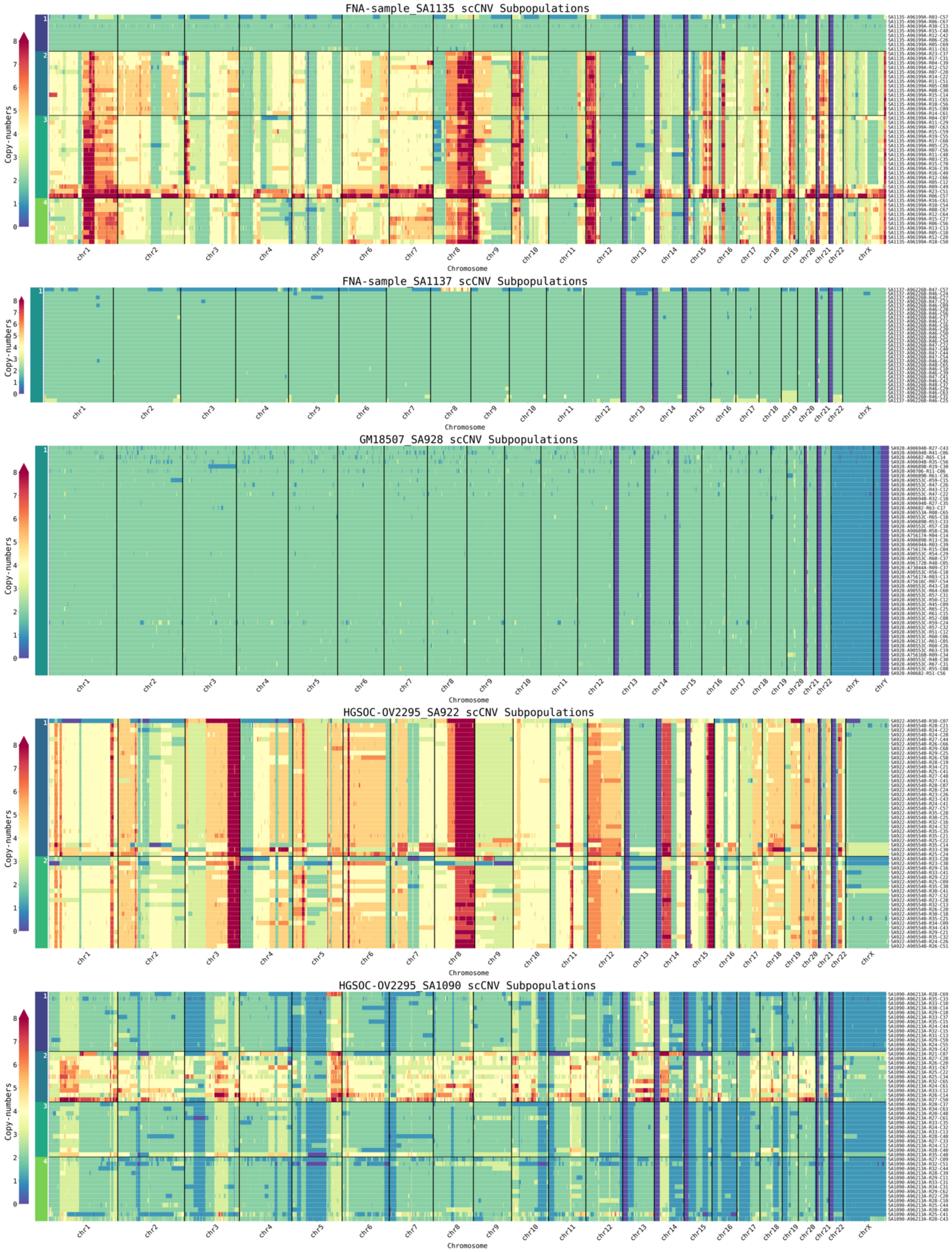
FNA-sample_SA1135 scCNV Subpopulations
FNA-sample_SA1137 scCNV Subpopulations
GM18507_SA928 scCNV Subpopulations
HGSOC-OV2295_SA922 scCNV Subpopulations
HGSOC-OV2295_SA1090 scCNV Subpopulations
Copy-numbers
Chromosome
chr1 chr2 chr3 chr4 chr5 chr6 chr7 chr8 chr9 chr10 chr11 chr12 chr13 chr14 chr15 chr16 chr17 chr18 chr19 chr20 chr21 chr22 chrX chrY

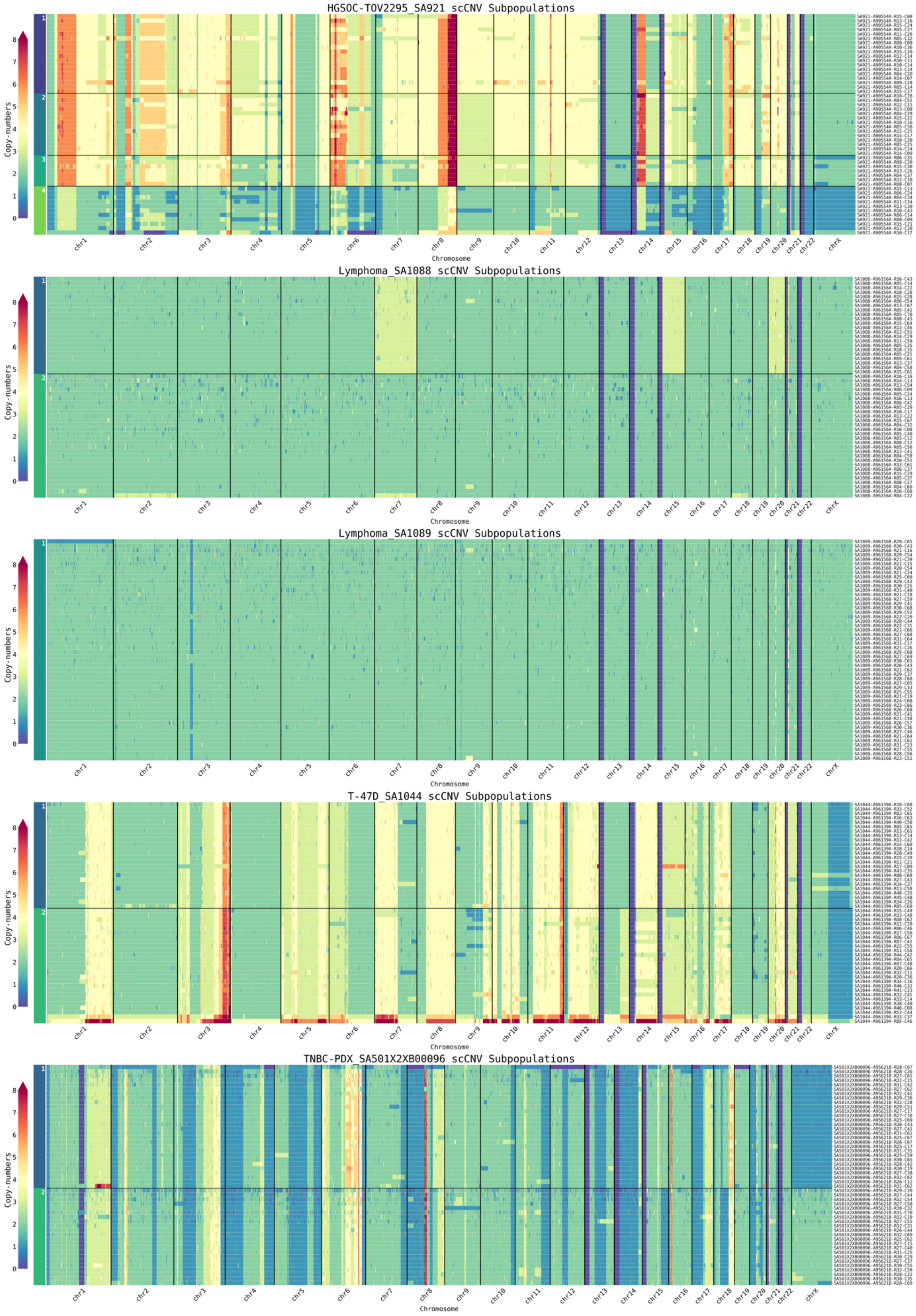
HGSOC-TOV2295_SA921 scCNV Subpopulations
Lymphoma_SA1088 scCNV Subpopulations
Lymphoma_SA1089 scCNV Subpopulations
T-47D_SA1044 scCNV Subpopulations
TNBC-PDX_SA501X2XB00096 scCNV Subpopulations
Copy-numbers
0
1
2
3
4
5
6
7
8
chr1
chr2
chr3
chr4
chr5
chr6
chr7
chr8
chr9
chr10
chr11
chr12
chr13
chr14
chr15
chr16
chr17
chr18
chr19
chr20
chr21
chr22
chrX
Chromosome

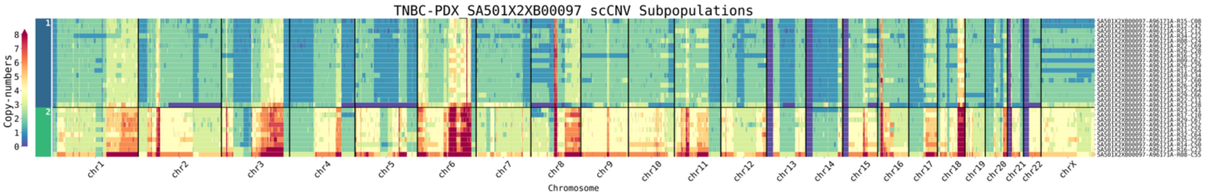
TNBC-PDX_SA501X2XB00097 scCNV Subpopulations
Copy-numbers
Chromosome

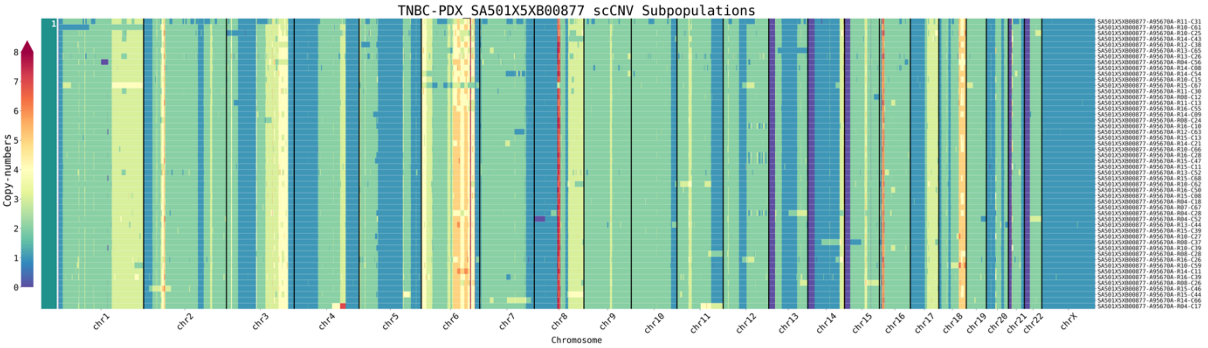
TNBC-PDX_SA501X5XB00877 scCNV Subpopulations
Copy-numbers
Chromosome

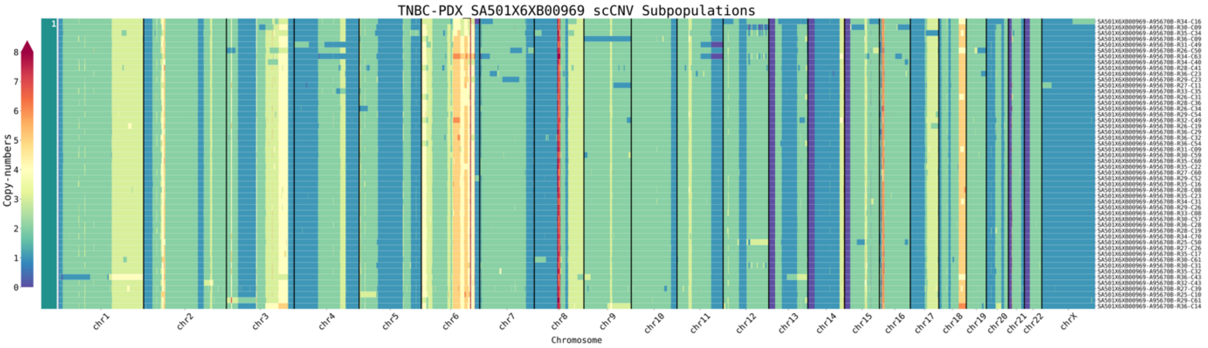
TNBC-PDX_SA501X6XB00969 scCNV Subpopulations
Copy-numbers
Chromosome

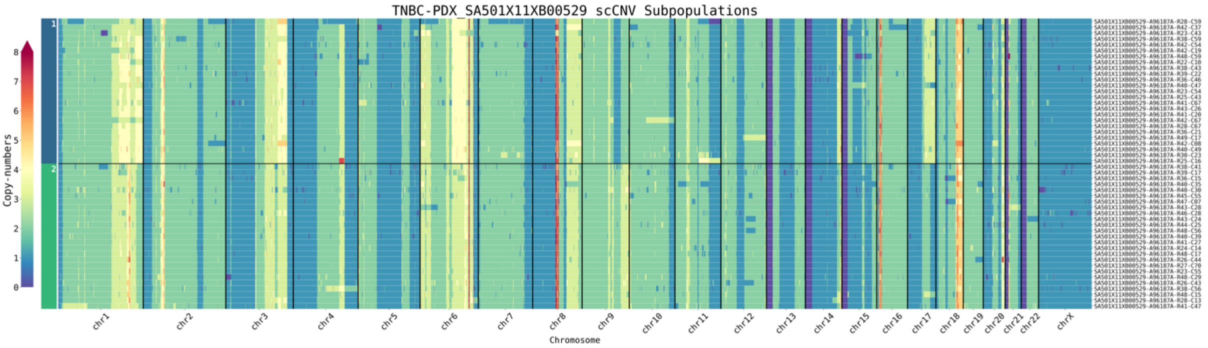
TNBC-PDX_SA501X11XB00529 scCNV Subpopulations
Copy-numbers
Chromosome

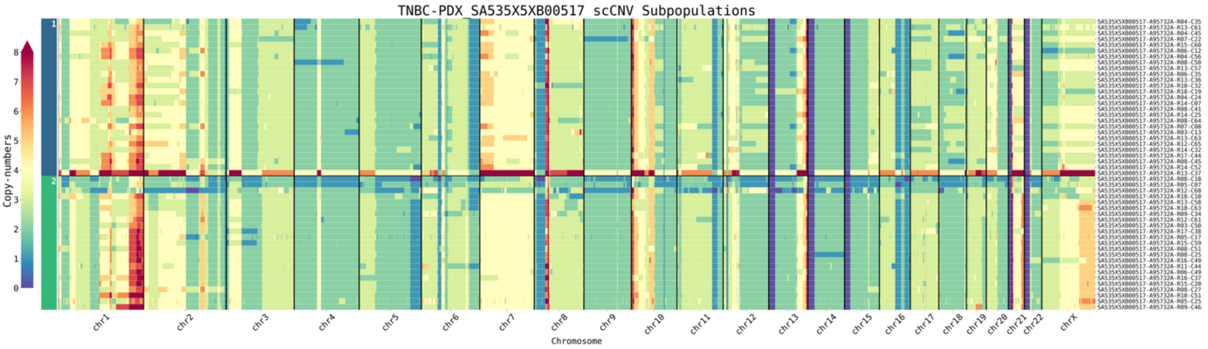
TNBC-PDX_SA535X5XB00517 scCNV Subpopulations
Copy-numbers
Chromosome

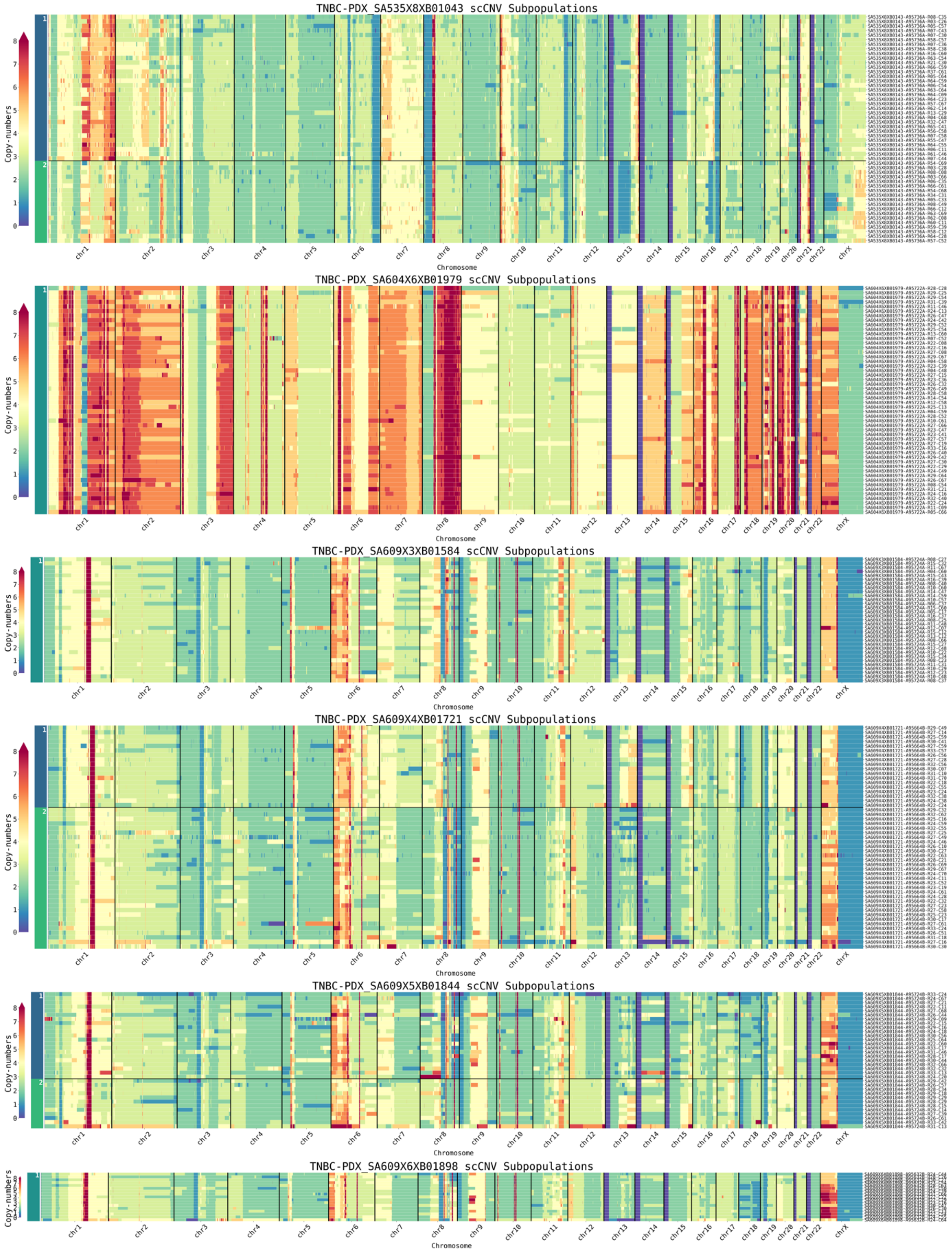
TNBC-PDX_SA535X8XB01043 scCNV Subpopulations
TNBC-PDX_SA604X6XB01979 scCNV Subpopulations
TNBC-PDX_SA609X3XB01584 scCNV Subpopulations
TNBC-PDX_SA609X4XB01721 scCNV Subpopulations
TNBC-PDX_SA609X5XB01844 scCNV Subpopulations
TNBC-PDX_SA609X6XB01898 scCNV Subpopulations
Copy-numbers
0
1
2
3
4
5
6
7
8
Chromosome
chr1
chr2
chr3
chr4
chr5
chr6
chr7
chr8
chr9
chr10
chr11
chr12
chr13
chr14
chr15
chr16
chr17
chr18
chr19
chr20
chr21
chr22
chrX

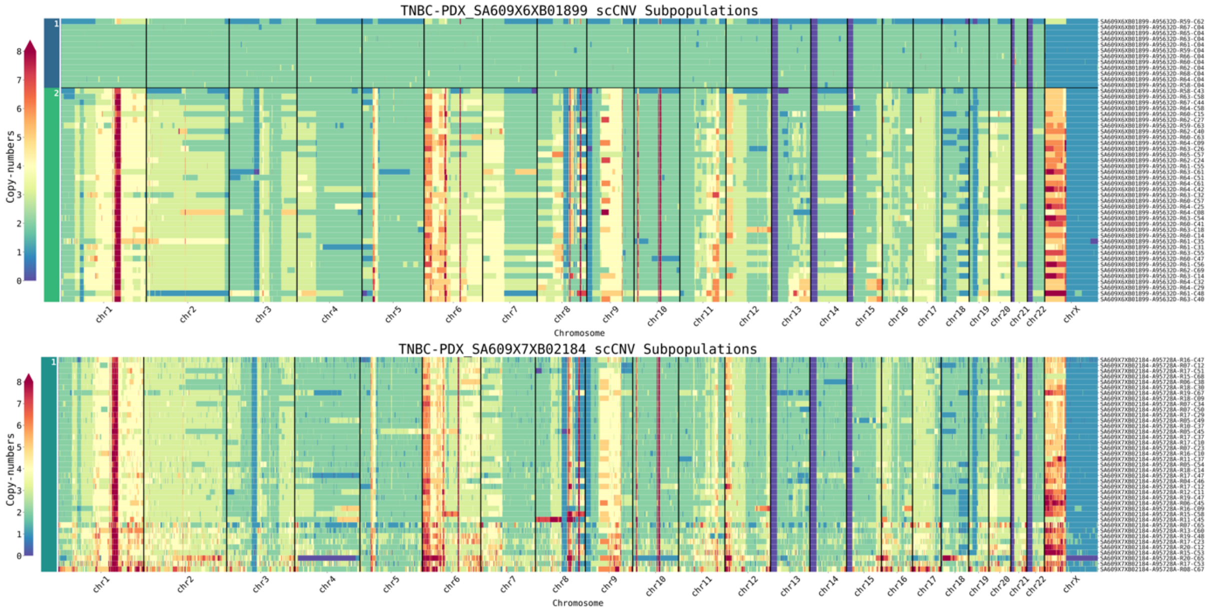
TNBC-PDX_SA609X6XB01899 scCNV Subpopulations
Copy-numbers
Chromosome
TNBC-PDX_SA609X7XB02184 scCNV Subpopulations
Copy-numbers
Chromosome

e

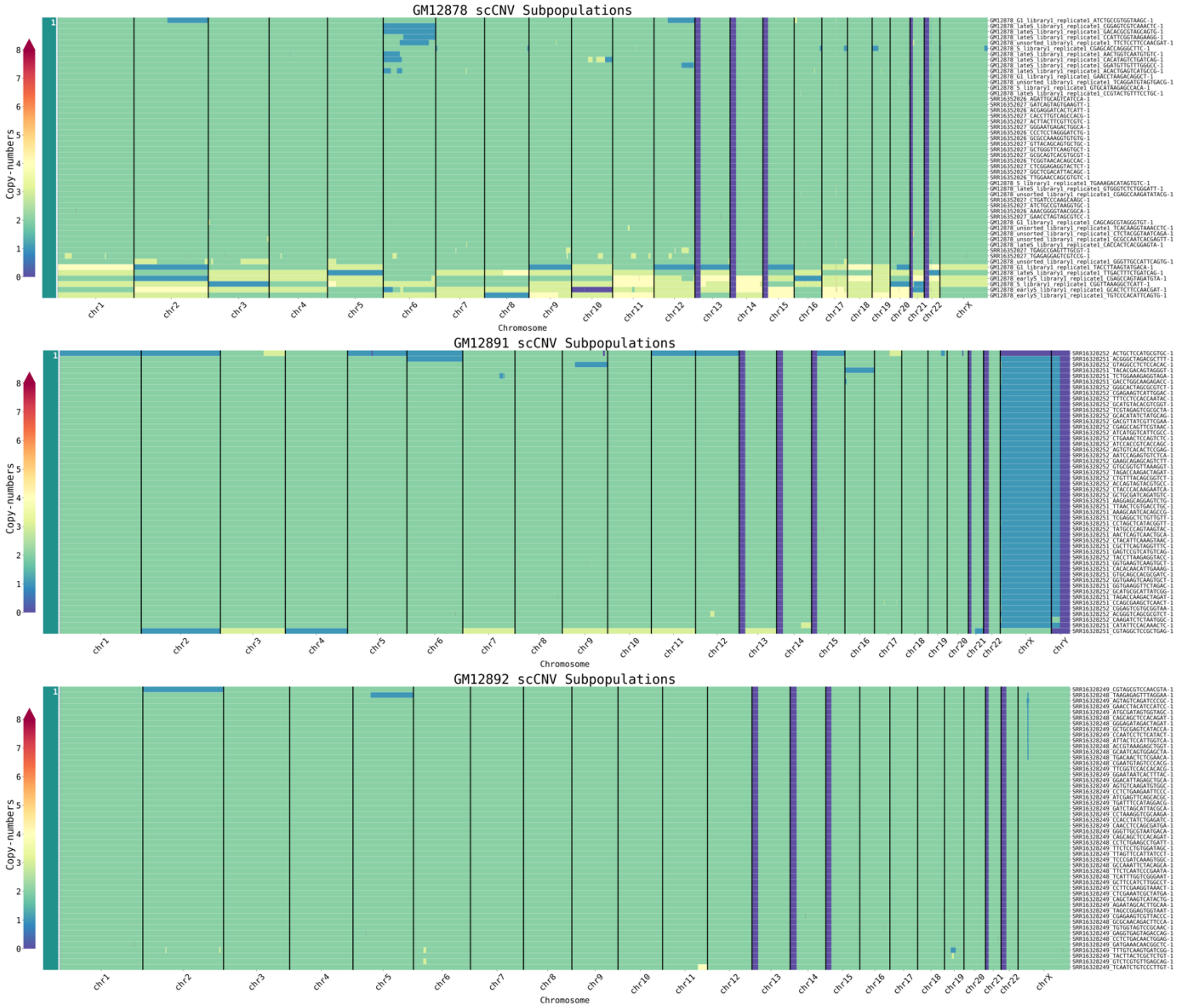
GM12878 scCNV Subpopulations
Copy-numbers
Chromosome
GM12891 scCNV Subpopulations
Copy-numbers
Chromosome
GM12892 scCNV Subpopulations
Copy-numbers
Chromosome

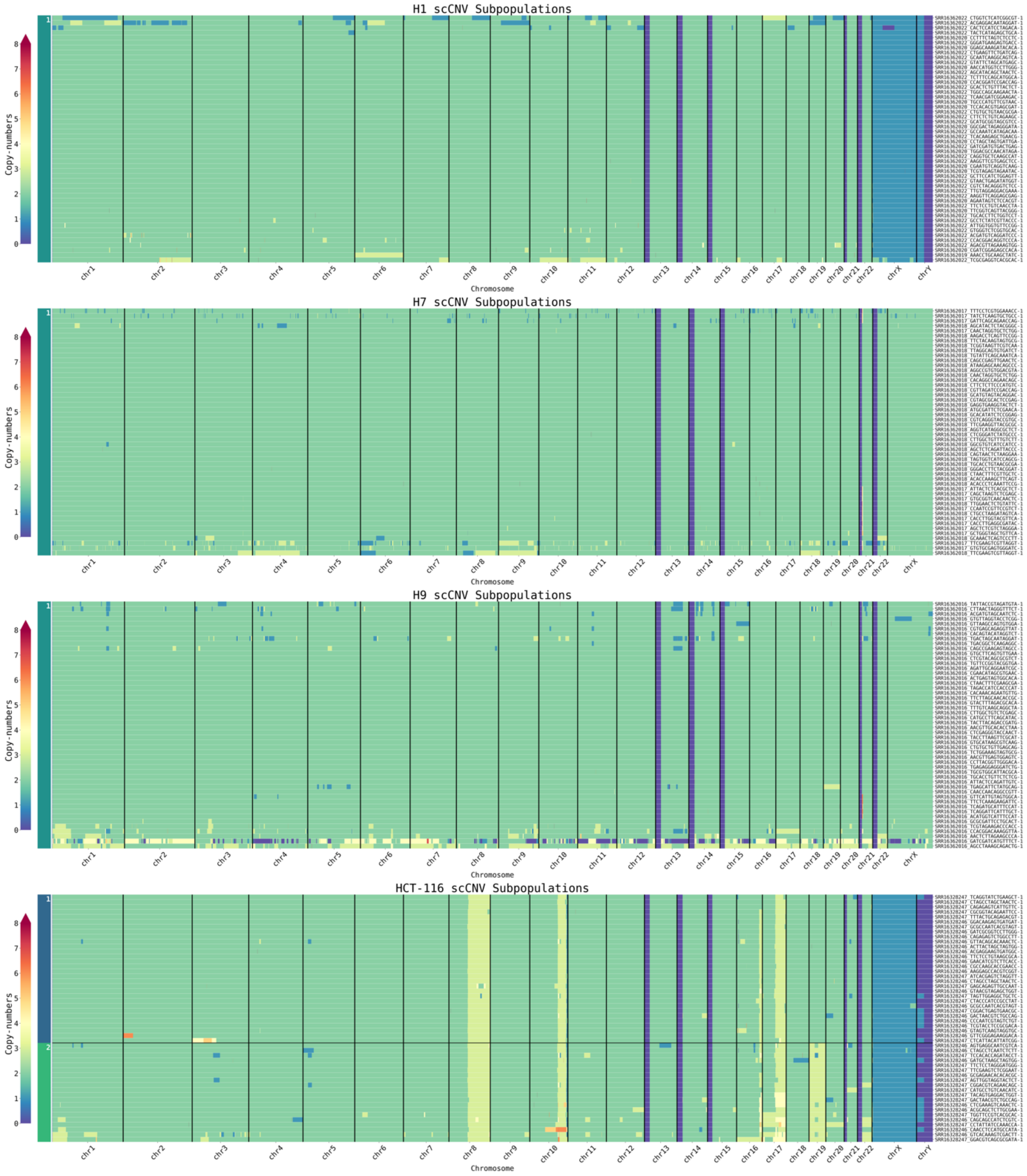
H1 scCNV Subpopulations
H7 scCNV Subpopulations
H9 scCNV Subpopulations
HCT-116 scCNV Subpopulations
Copy-numbers
0
1
2
3
4
5
6
7
8
chr1
chr2
chr3
chr4
chr5
chr6
chr7
chr8
chr9
chr10
chr11
chr12
chr13
chr14
chr15
chr16
chr17
chr18
chr19
chr20
chr21
chr22
chrX
chrY
Chromosome

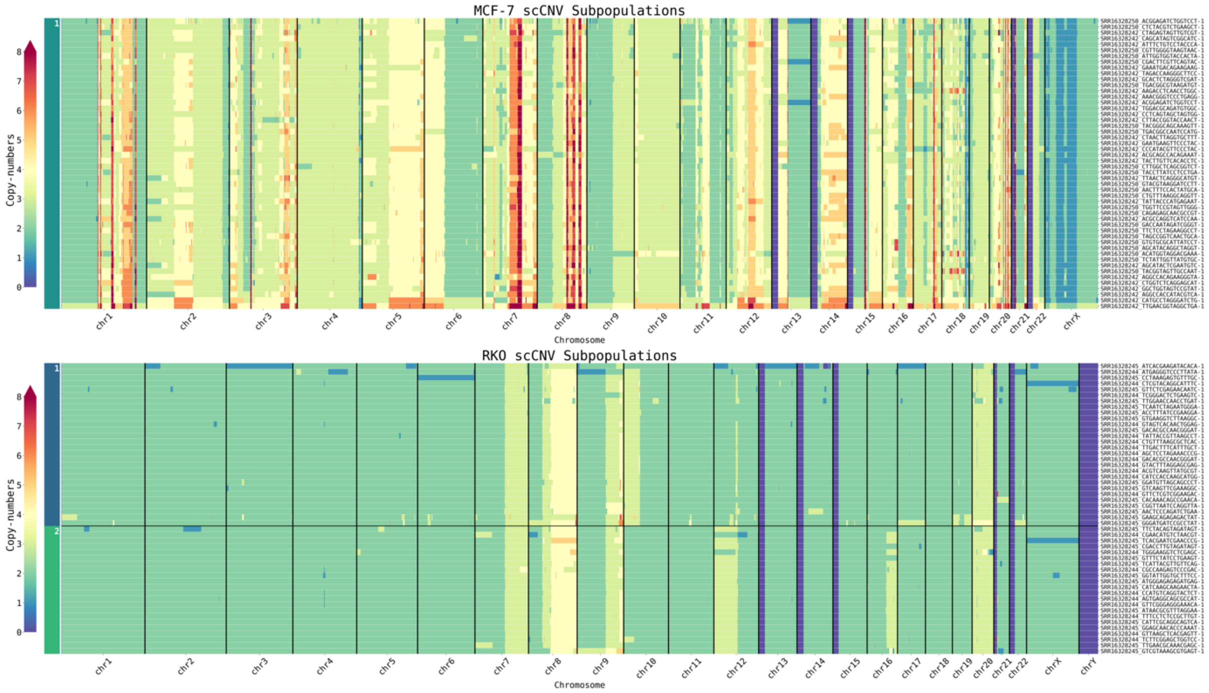
MCF-7 scCNV Subpopulations
Copy-numbers
Chromosome
RKO scCNV Subpopulations
Copy-numbers
Chromosome

f

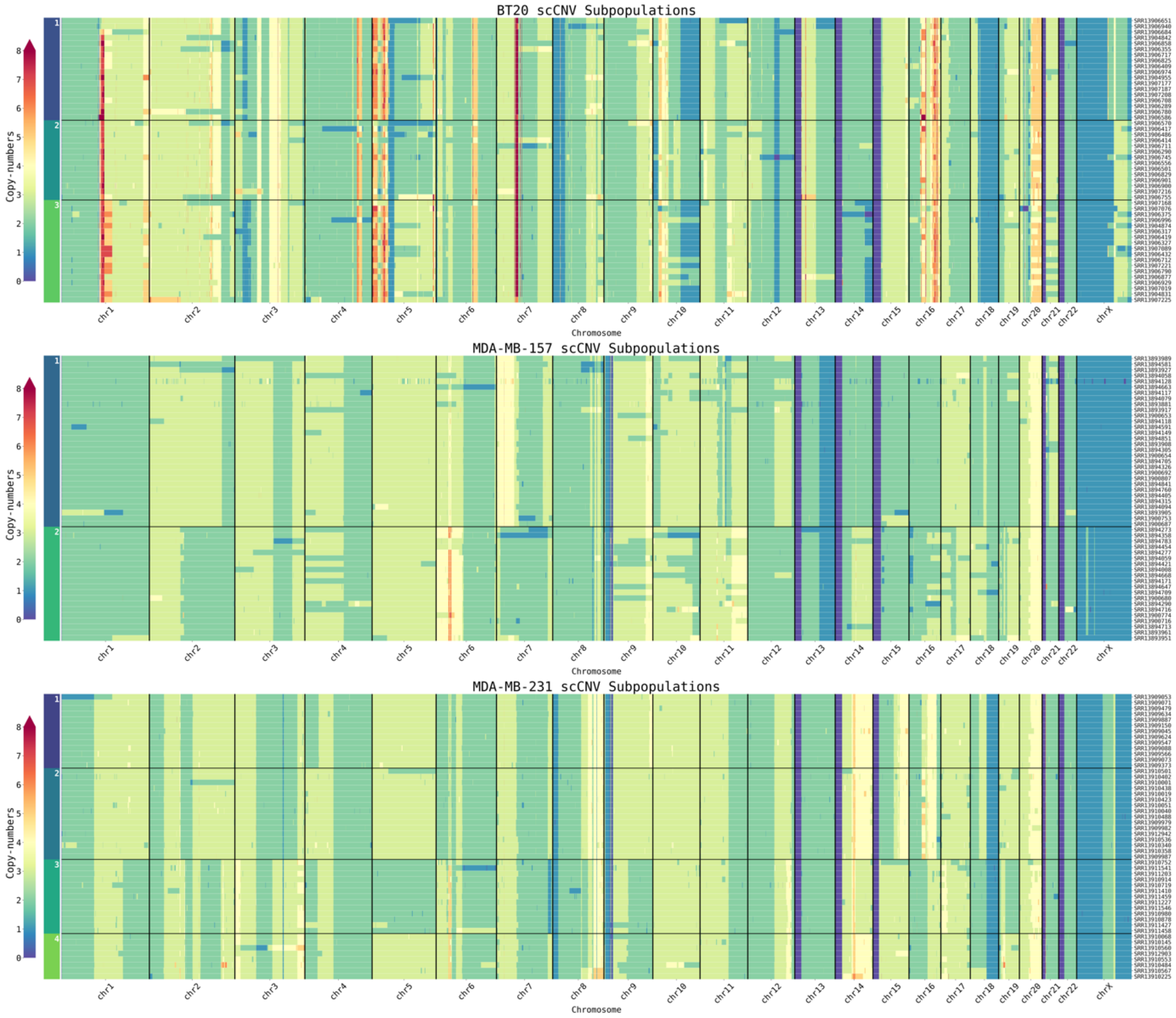
BT20 scCNV Subpopulations
Copy-numbers
Chromosome
MDA-MB-157 scCNV Subpopulations
Copy-numbers
Chromosome
MDA-MB-231 scCNV Subpopulations
Copy-numbers
Chromosome

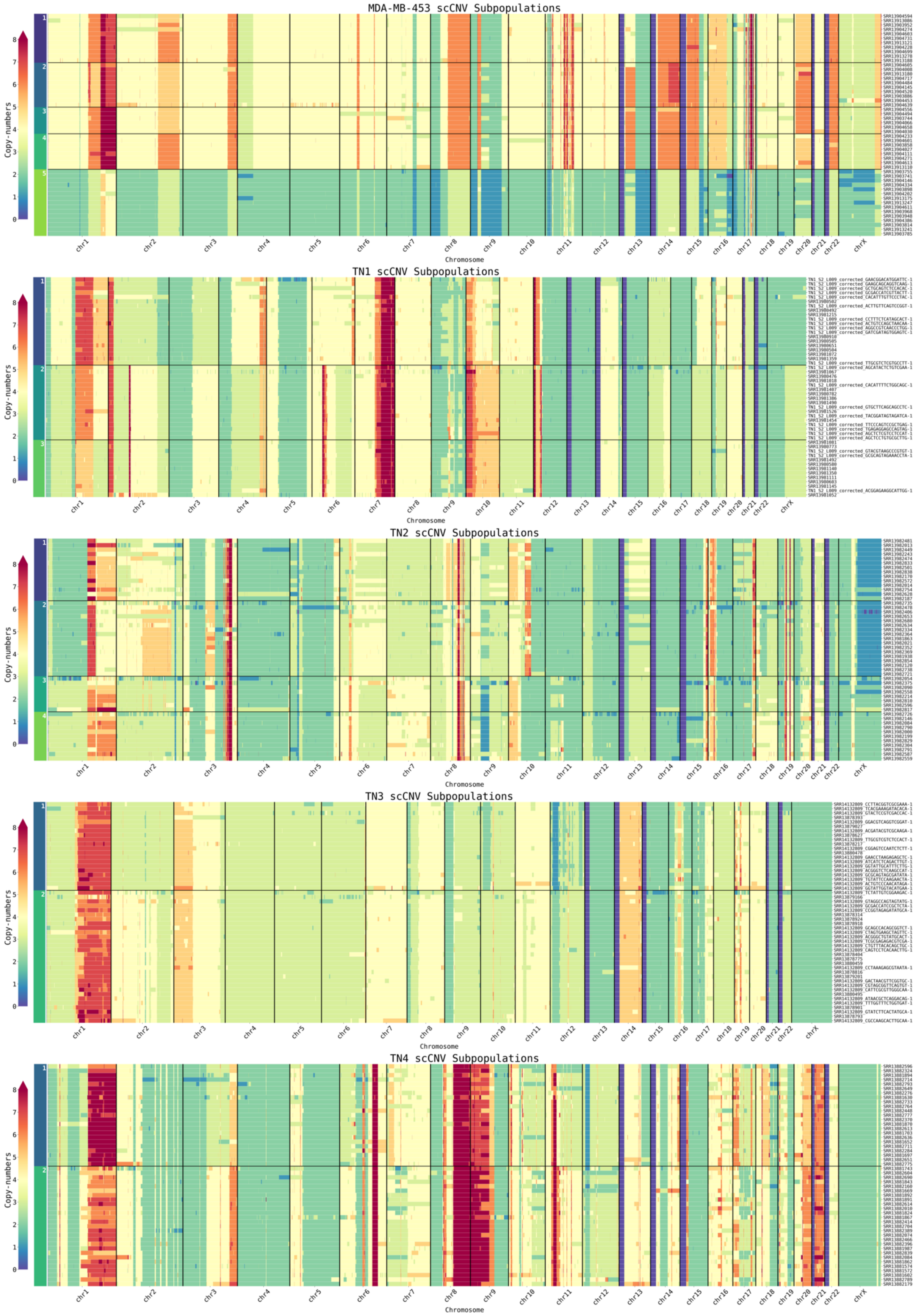
MDA-MB-453 scCNV Subpopulations
TN1 scCNV Subpopulations
TN2 scCNV Subpopulations
TN3 scCNV Subpopulations
TN4 scCNV Subpopulations
Copy-numbers
0
1
2
3
4
5
6
7
8
chr1
chr2
chr3
chr4
chr5
chr6
chr7
chr8
chr9
chr10
chr11
chr12
chr13
chr14
chr15
chr16
chr17
chr18
chr19
chr20
chr21
chr22
chrX
Chromosome

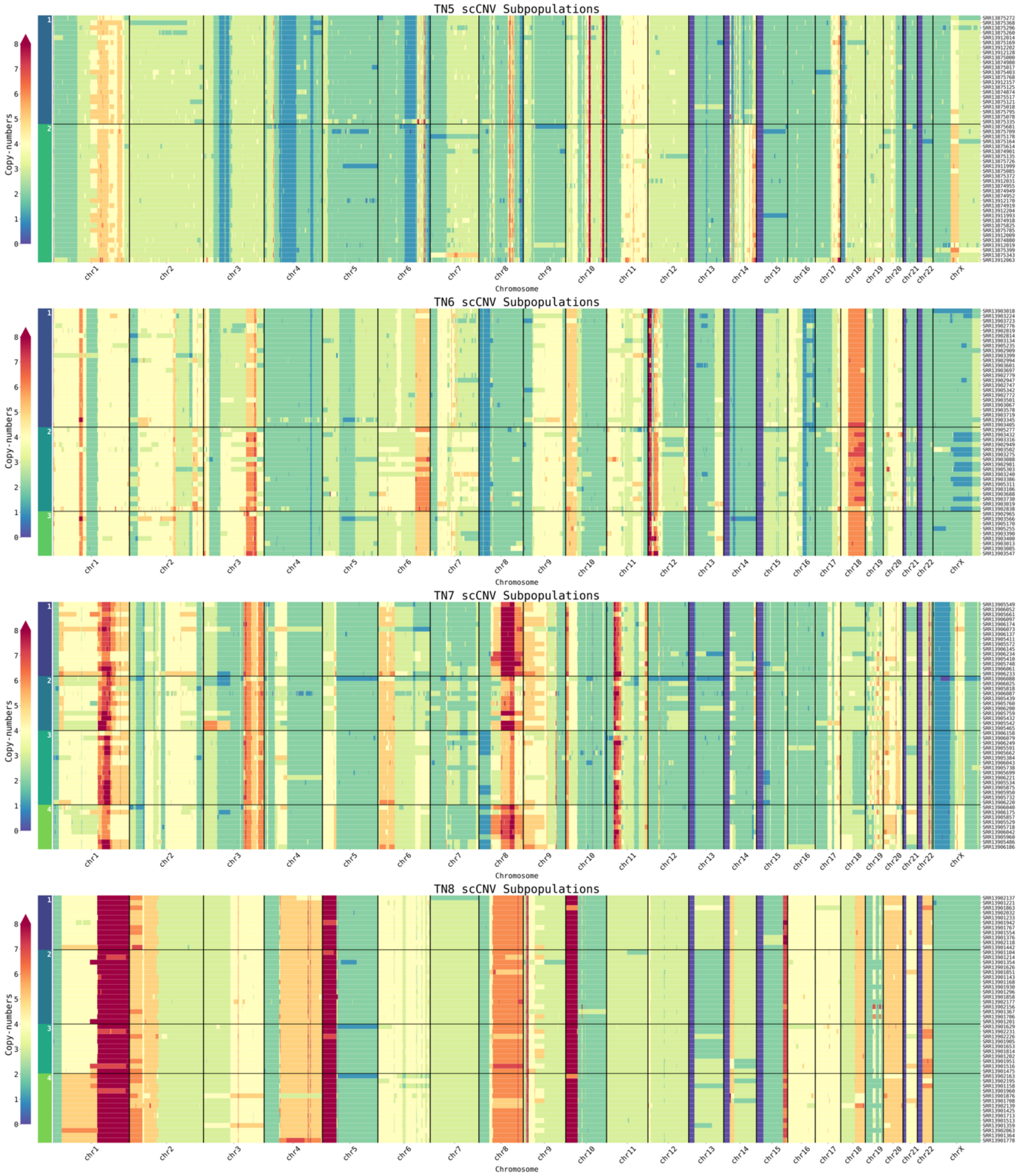
TN5 scCNV Subpopulations
TN6 scCNV Subpopulations
TN7 scCNV Subpopulations
TN8 scCNV Subpopulations
Copy-numbers
Chromosome
chr1 chr2 chr3 chr4 chr5 chr6 chr7 chr8 chr9 chr10 chr11 chr12 chr13 chr14 chr15 chr16 chr17 chr18 chr19 chr20 chr21 chr22 chrX

g

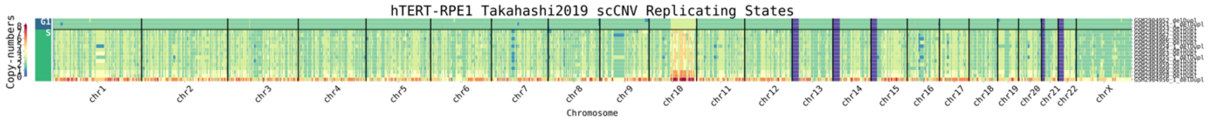
hTERT-RPE1 Takahashi2019 scCNV Replicating States
Copy-numbers
G1
S
Chromosome
chr1 chr2 chr3 chr4 chr5 chr6 chr7 chr8 chr9 chr10 chr11 chr12 chr13 chr14 chr15 chr16 chr17 chr18 chr19 chr20 chr21 chr22 chrX

h

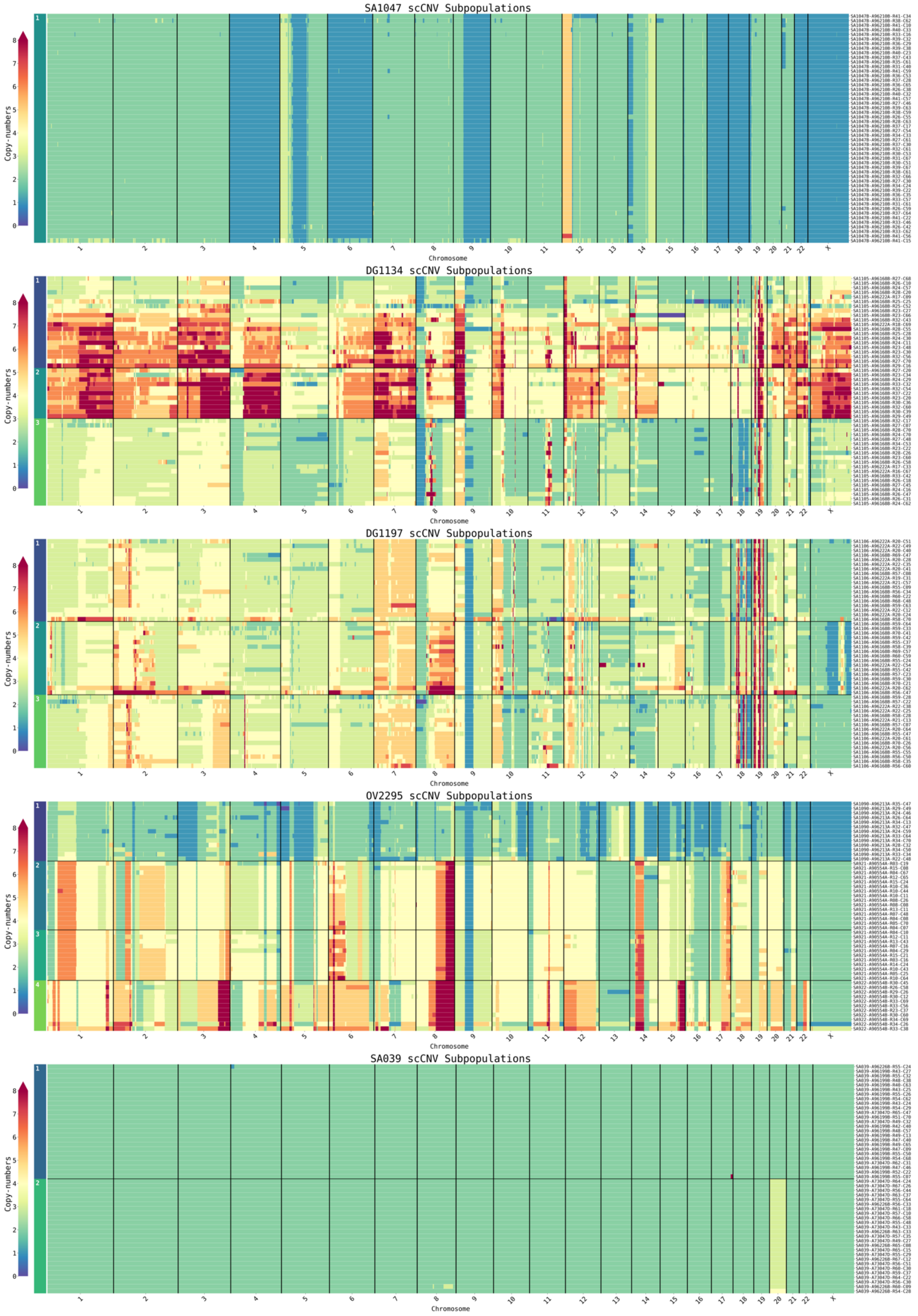
SA1047 scCNV Subpopulations
DG1134 scCNV Subpopulations
DG1197 scCNV Subpopulations
OV2295 scCNV Subpopulations
SA039 scCNV Subpopulations
Copy-numbers
0
1
2
3
4
5
6
7
8
Chromosome
1 2 3 4 5 6 7 8 9 10 11 12 13 14 15 16 17 18 19 20 21 22 X

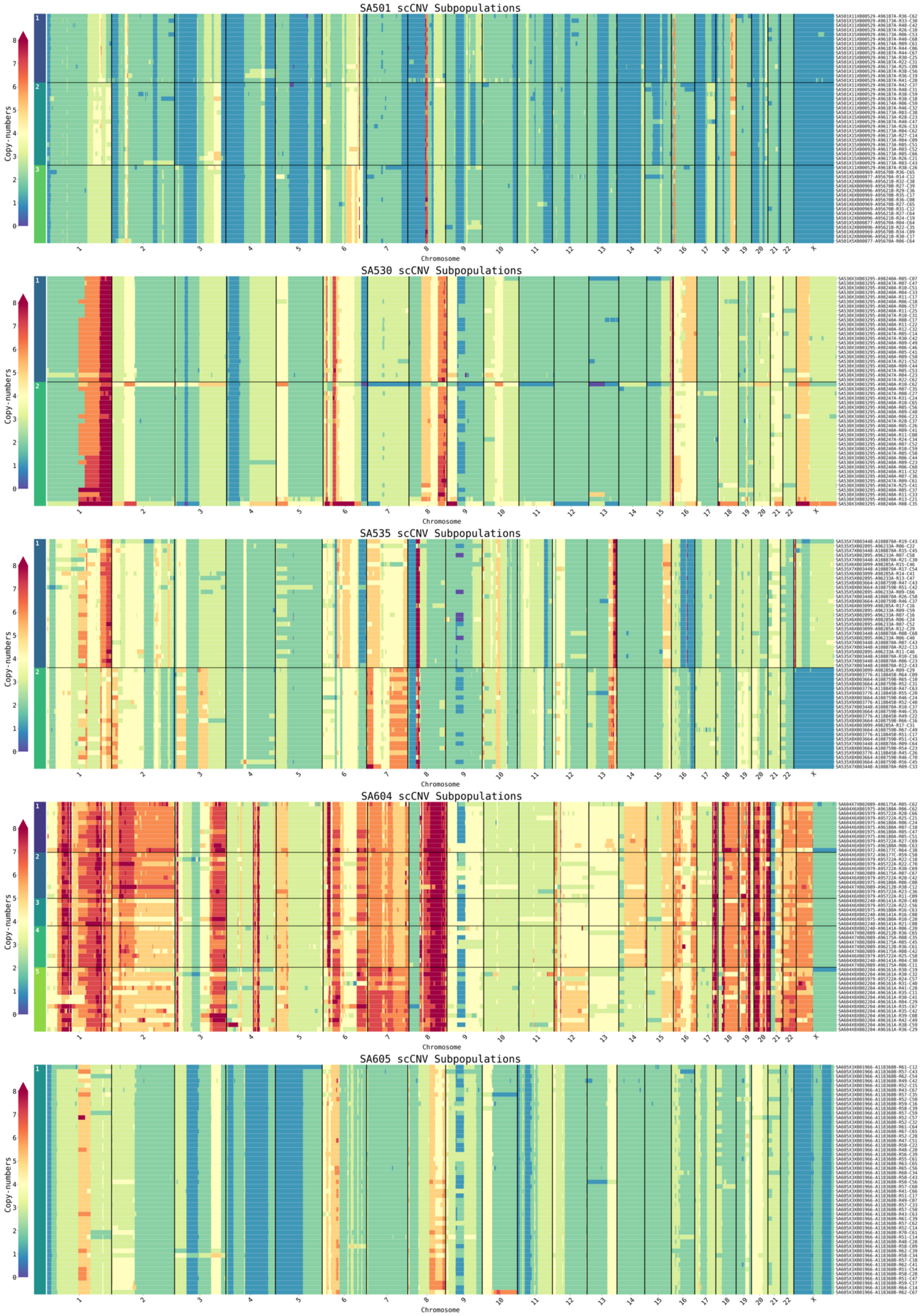
SA501 scCNV Subpopulations
SA530 scCNV Subpopulations
SA535 scCNV Subpopulations
SA604 scCNV Subpopulations
SA605 scCNV Subpopulations
Copy-numbers
Chromosome
1 2 3 4 5 6 7 8 9 10 11 12 13 14 15 16 17 18 19 20 21 22 X

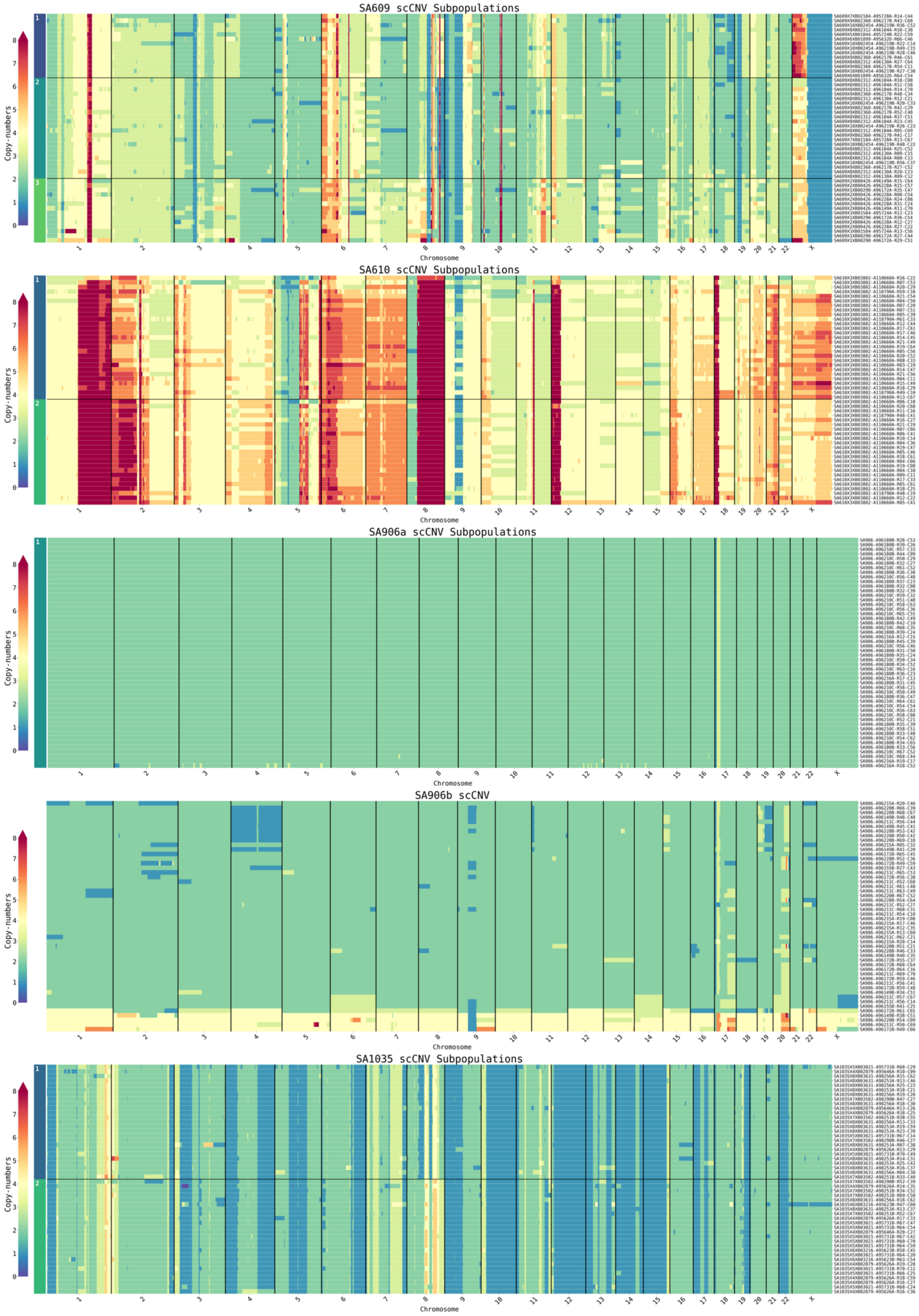

SA609 scCNV Subpopulations
SA610 scCNV Subpopulations
SA906a scCNV Subpopulations
SA906b scCNV
SA1035 scCNV Subpopulations
Copy-numbers
Chromosome

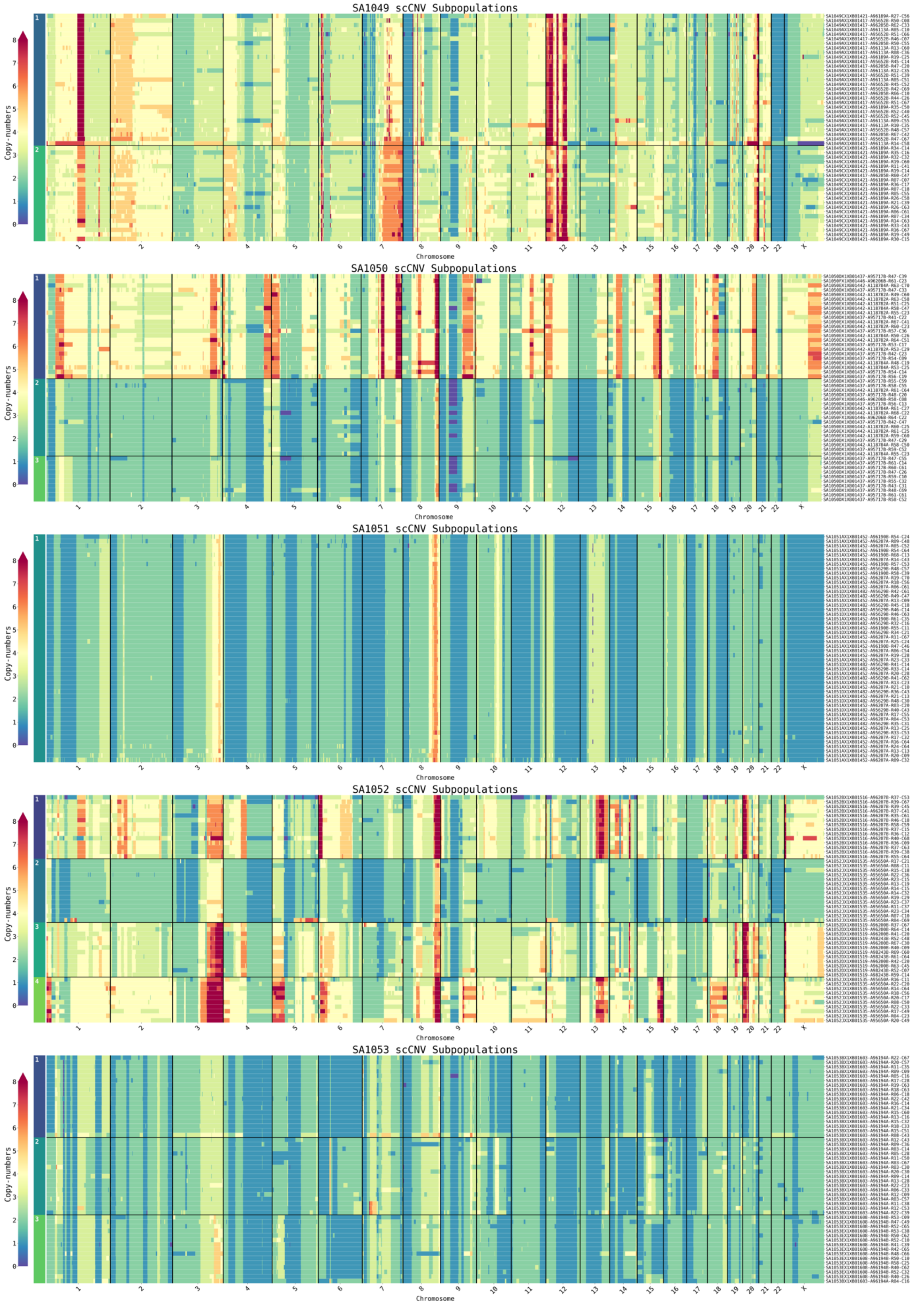

SA1049 scCNV Subpopulations
SA1050 scCNV Subpopulations
SA1051 scCNV Subpopulations
SA1052 scCNV Subpopulations
SA1053 scCNV Subpopulations
Copy-numbers
Chromosome

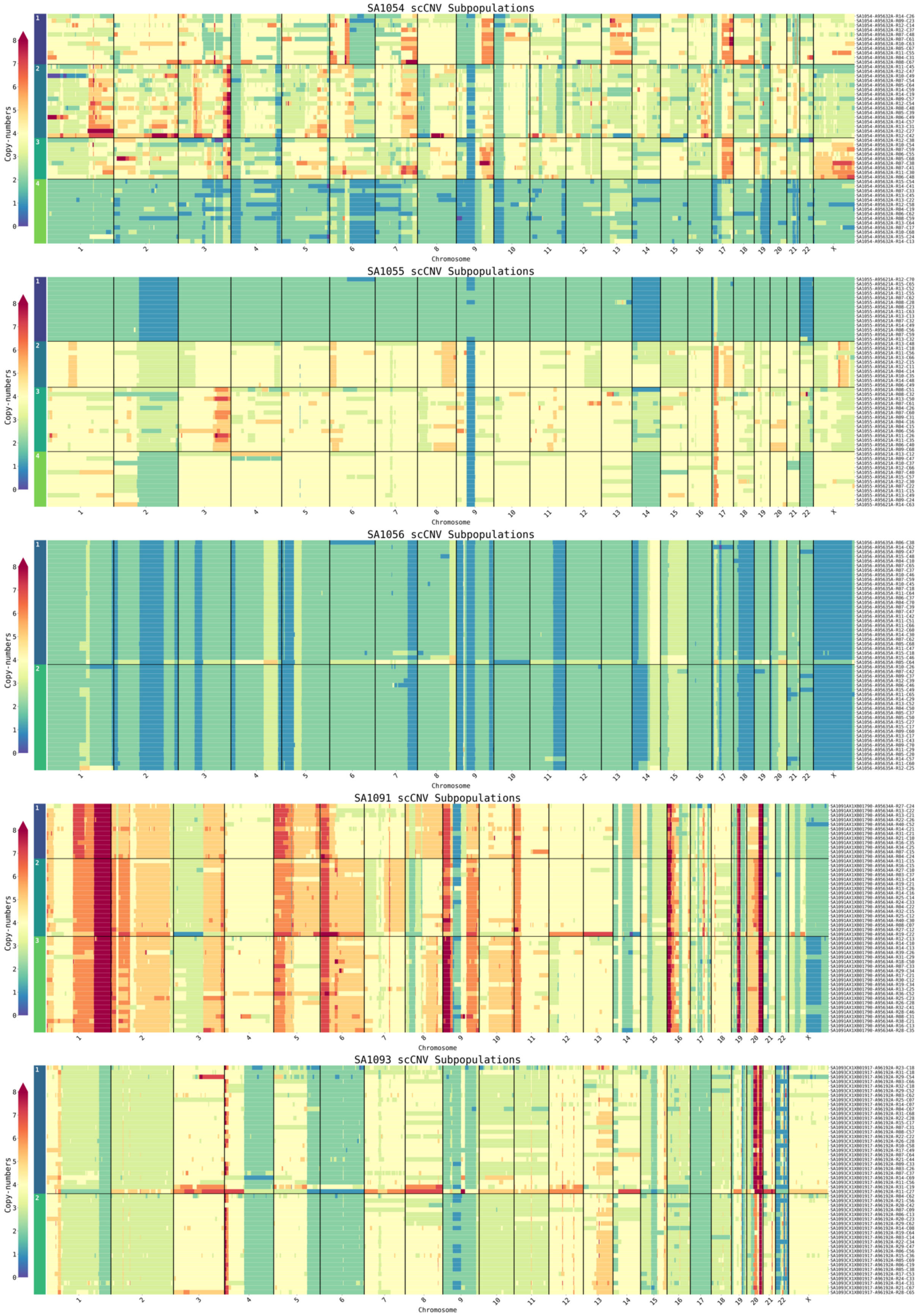
SA1054 scCNV Subpopulations
Copy-numbers
Chromosome
SA1055 scCNV Subpopulations
Copy-numbers
Chromosome
SA1056 scCNV Subpopulations
Copy-numbers
Chromosome
SA1091 scCNV Subpopulations
Copy-numbers
Chromosome
SA1093 scCNV Subpopulations
Copy-numbers
Chromosome

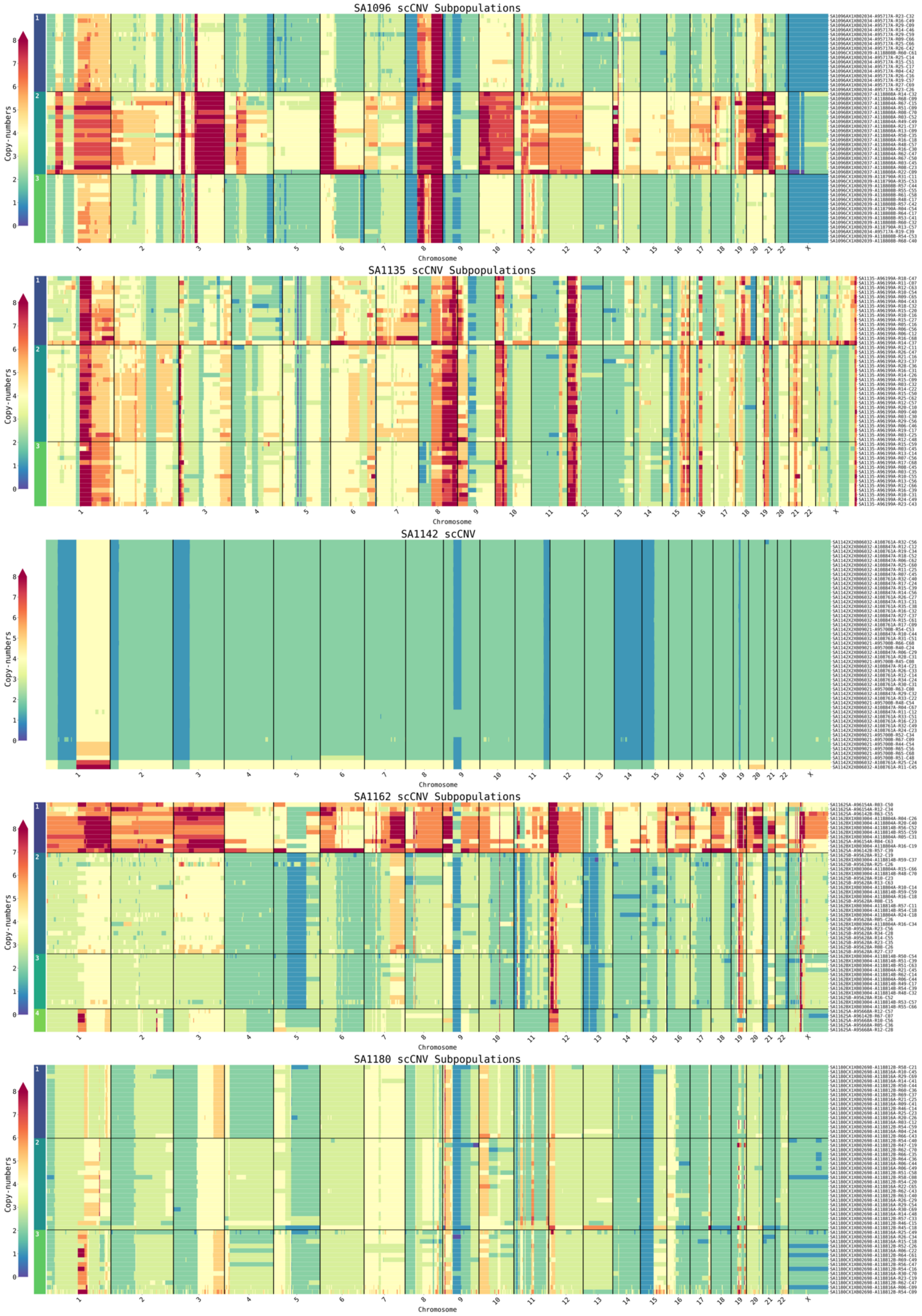

SA1096 scCNV Subpopulations
SA1135 scCNV Subpopulations
SA1142 scCNV
SA1162 scCNV Subpopulations
SA1180 scCNV Subpopulations
Copy-numbers
Chromosome
1
2
3
4
5
6
7
8
9
10
11
12
13
14
15
16
17
18
19
20
21
22
X

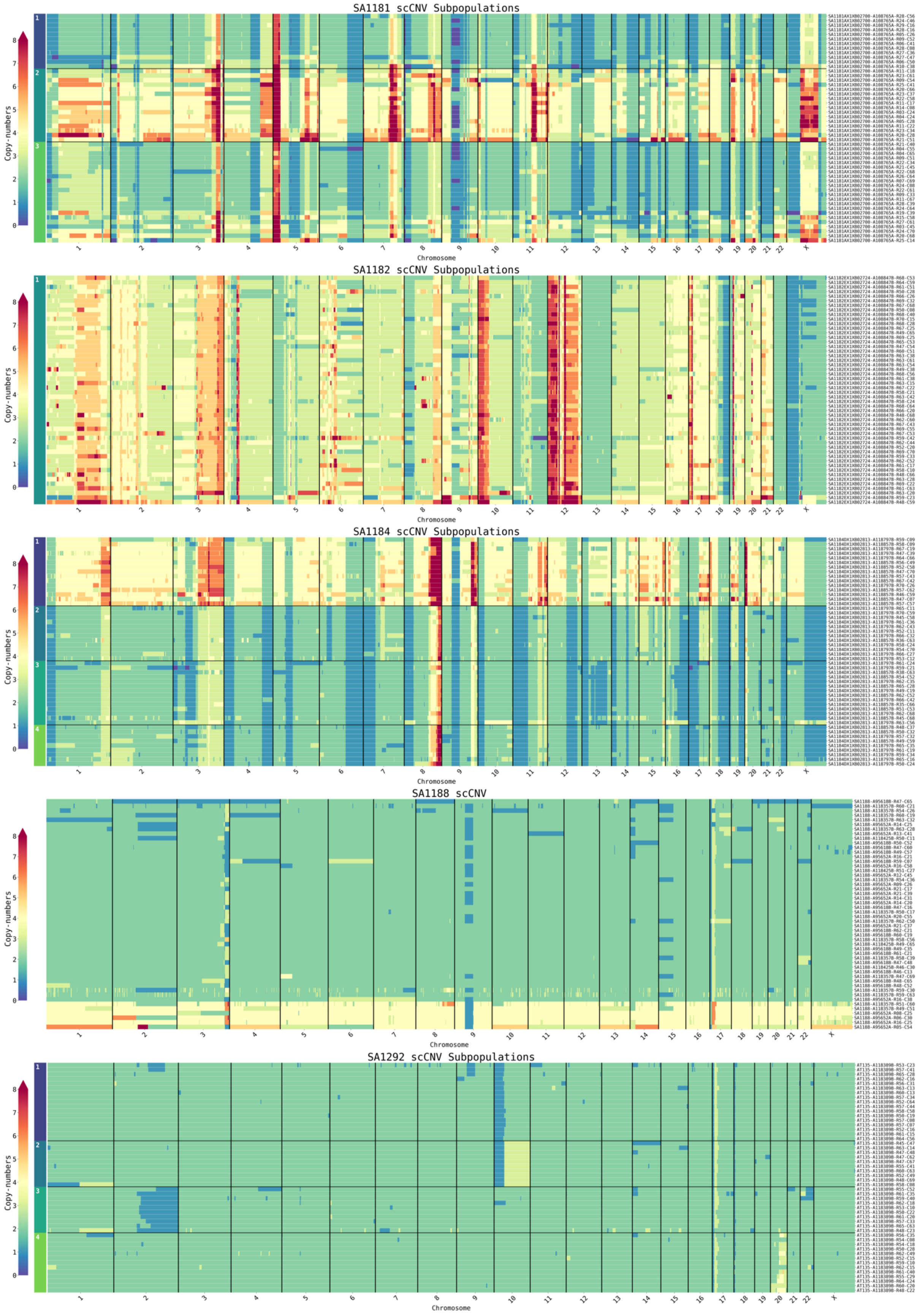

Supplementary Figure S3. Genome-wide scCNV profiles. A maximum of 50 randomly selected cells for Connolly 2022 (a), Du 2021 (b), Gnan 2022 (c), Laks 2019 (d), Massey 2022 (e), Minussi 2021 (f), Takahashi 2019 (g) and Funnell 2022 (h) split by cell-phase (a, g) or subpopulation (b-f, h) and coloured by DNA copy numbers. Source data are provided as a Source Data file.

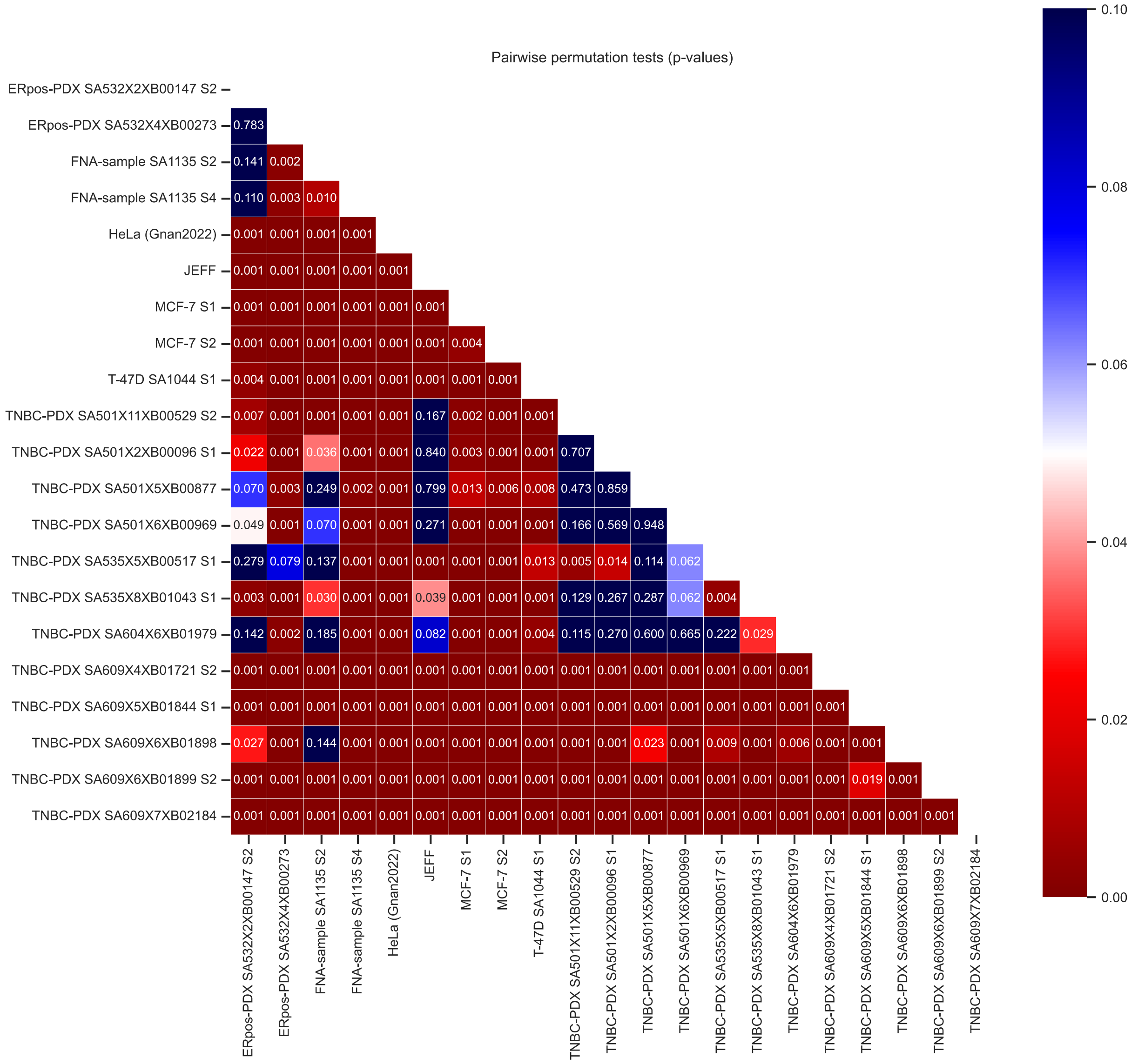

Supplementary Figure S4. Pairwise permutation test p-values from the scRT trajectories. Pairwise permutation test p-values are shown for single-cell replication timing (scRT) trajectories from Figure 7b, with significant values ($p < 0.05$) highlighted in shades of red. These p-values reflect the observed correlations between replication timing (RT) profiles from the scRT atlas (Figure 6) and allow determining whether the RT has changed between subpopulations and/or PDX passages. Source data are provided as a Source Data file.

Supplementary Table S1. The number and percentage of missing copy number values of the data processed in this study.

Dataset	Cell type	Missing values	% of missing values
Du2021	HCT116 DKO1	82572	0.42%
Du2021	HCT116 WT	77757	0.37%
Gnan2022	HeLa	139581	0.84%
Gnan2022	JEFF	232765	0.68%
Gnan2022	MCF-7	734532	1.11%
Laks2019	184-hTERT_SA039	112150	0.11%
Laks2019	184-hTERT_SA1101	66397	0.16%
Laks2019	184-hTERT_SA906	422382	0.21%
Laks2019	ERpos-PDX_SA532X2XB00147	47774	0.74%
Laks2019	ERpos-PDX_SA532X4XB00273	83231	0.67%
Laks2019	ERpos-PDX_SA532X8XB01398	47633	0.75%
Laks2019	ERpos-PDX_SA611X3XB00821	6392	0.11%
Laks2019	ERpos-PDX_SA995X5XB01910	9903	0.37%
Laks2019	FNA-sample_SA1135	90556	0.89%
Laks2019	FNA-sample_SA1137	164	0.02%
Laks2019	GM18507_SA928	478625	0.30%
Laks2019	HGSOC-OV2295_SA1090	38441	0.21%
Laks2019	HGSOC-OV2295_SA922	12378	0.26%
Laks2019	HGSOC-TOV2295_SA921	6708	0.18%
Laks2019	HeLa_SA1087	59481	0.51%
Laks2019	Lymphoma_SA1088	7298	0.09%
Laks2019	Lymphoma_SA1089	9969	0.11%
Laks2019	T-47D_SA1044	137877	0.41%
Laks2019	TNBC-PDX_SA501X11XB00529	30923	0.14%
Laks2019	TNBC-PDX_SA501X2XB00096	100404	0.76%
Laks2019	TNBC-PDX_SA501X2XB00097	6338	0.72%
Laks2019	TNBC-PDX_SA501X5XB00877	19919	0.31%
Laks2019	TNBC-PDX_SA501X6XB00969	7524	0.09%
Laks2019	TNBC-PDX_SA535X5XB00517	30467	0.29%
Laks2019	TNBC-PDX_SA535X8XB01043	29907	0.36%
Laks2019	TNBC-PDX_SA604X6XB01979	70601	0.67%
Laks2019	TNBC-PDX_SA609X3XB01584	16072	0.45%
Laks2019	TNBC-PDX_SA609X4XB01721	42339	0.41%
Laks2019	TNBC-PDX_SA609X5XB01844	27802	0.31%
Laks2019	TNBC-PDX_SA609X6XB01898	23087	0.25%
Laks2019	TNBC-PDX_SA609X6XB01899	35915	0.30%
Laks2019	TNBC-PDX_SA609X7XB02184	42705	0.58%
Massey2022	GM12878	106746	0.07%
Massey2022	GM12891	9197	0.02%
Massey2022	GM12892	30559	0.06%
Massey2022	H1	1445	0.01%
Massey2022	H7	21129	0.06%
Massey2022	H9	2284	0.01%
Massey2022	HCT-116	3485	0.01%
Massey2022	MCF-7	33682	0.22%
Massey2022	RKO	13795	0.03%
Minussi2021	BT20	399381	1.11%
Minussi2021	MDA-MB-157	64828	0.18%
Minussi2021	MDA-MB-231	430042	0.54%
Minussi2021	MDA-MB-453	256514	0.69%
Minussi2021	TN1	555575	0.95%
Minussi2021	TN2	468262	1.56%
Minussi2021	TN3	311615	0.48%
Minussi2021	TN4	363962	0.95%
Minussi2021	TN5	256631	0.71%
Minussi2021	TN6	100736	0.28%
Minussi2021	TN7	69698	0.26%
Minussi2021	TN8	278128	0.77%
Connolly2022	hTERT-RPE1	11014	0.60%
Takahashi2019	hTERT-RPE1	4352	0.87%
		MEDIAN	0.34%
		MEAN	0.43%
		MAX	1.56%
		MIN	0.01%

Supplementary Table S2. Data used for the single-cell DNA replication state classifier step.

Dataset	Cell type	Cell cycle phase	Cell count
Massey2022	GM12878	G1	1193
Massey2022	GM12878	S	1655
Du2021	HCT116	G1	49
Du2021	HCT116	S	434
Du2021	HCT116_DKO1	G1	40
Du2021	HCT116_DKO1	S	338
Gnan2022	HeLa	G1	224
Gnan2022	HeLa	S	299
Gnan2022	JEFF	G1	132
Gnan2022	JEFF	S	998
Gnan2022	MCF7	G1	632
Gnan2022	MCF7	S	1512
Takahashi2019	hTERT-RPE1	G1	3
Takahashi2019	hTERT-RPE1	S	14

Supplementary Table S3. Quality metrics of the data used in this study. QC: Quality-Control.

Source	Sample	Pre-QC cell count	Post-QC Cell count	Percentage of cells - QC loss	Cell type	Cell type description	Sample type	Median coverage [reads/Mb]
Du2021	HCT116 DKO1	669	668	0.15%	HCT-116	Colon cancer	Cell line	1,882.02
Du2021	HCT116 WT	713	713	0.00%	HCT-116	Colon cancer	Cell line	1,176.35
Gnan2022	HeLa	752	752	0.00%	HeLa	Cervical carcinoma	Cell line	788.81
Gnan2022	JEFF	1,461	1,455	0.41%	JEFF	Lymphocyte	Cell line	425.48
Gnan2022	MCF-7	2,768	2,768	0.00%	MCF-7	Breast cancer	Cell line	720.07
Laks2019	184-hTERT_SA039	5,290	5,285	0.09%	184-hTERT	Mammary epithelial	Cell line	344.11
Laks2019	184-hTERT_SA1101	3,358	2,788	16.97%	184-hTERT	Mammary epithelial	Cell line	272.89
Laks2019	184-hTERT_SA906	12,707	10,695	15.83%	184-hTERT	Mammary epithelial	Cell line	475.97
Laks2019	ERpos-PDX_SA532X2XB00147	755	441	41.59%	PDX	Breast cancer	PDX	688.52
Laks2019	ERpos-PDX_SA532X4XB00273	635	498	21.57%	PDX	Breast cancer	PDX	404.47
Laks2019	ERpos-PDX_SA532X8XB01398	589	371	37.01%	PDX	Breast cancer	PDX	716.82
Laks2019	ERpos-PDX_SA611X3XB00821	531	436	17.89%	PDX	Breast cancer	PDX	417.50
Laks2019	ERpos-PDX_SA995X5XB01910	465	152	67.31%	PDX	Breast cancer	PDX	571.08
Laks2019	FNA-sample_SA1135	800	473	40.88%	Tumour	Breast cancer	Patient tumour	982.93
Laks2019	FNA-sample_SA1137	88	37	57.95%	Tumour	Breast cancer	Patient tumour	105.54
Laks2019	GM18507_SA928	8,218	7,461	9.21%	GM18507	Lymphocyte	Cell line	678.99
Laks2019	HeLa_SA1087	656	601	8.38%	HeLa	Cervical carcinoma	Cell line	416.94
Laks2019	HGSOC-OV2295_SA1090	741	696	6.07%	OV2295	HGSC	Cell line	596.04
Laks2019	HGSOC-OV2295_SA922	1,085	368	66.08%	OV2295	HGSC	Cell line	849.26
Laks2019	HGSOC-TOV2295_SA921	1,118	371	66.82%	TOV2295	HGSC	Cell line	868.14
Laks2019	Lymphoma_SA1088	648	530	18.21%	Lymphoma	Follicular lymphoma	Patient tumour	459.55
Laks2019	Lymphoma_SA1089	375	346	7.73%	Lymphoma	Follicular lymphoma	Patient tumour	620.18
Laks2019	T-47D_SA1044	1,436	1,332	7.24%	T-47	Breast cancer	Cell line	1,021.92
Laks2019	TNBC-PDX_SA501X11XB00529	1,063	954	10.25%	PDX	Breast cancer	PDX	233.80
Laks2019	TNBC-PDX_SA501X2XB00096	488	451	7.58%	PDX	Breast cancer	PDX	1,335.05
Laks2019	TNBC-PDX_SA501X2XB00097	492	37	92.48%	PDX	Breast cancer	PDX	733.97
Laks2019	TNBC-PDX_SA501X5XB00877	615	270	56.10%	PDX	Breast cancer	PDX	364.75
Laks2019	TNBC-PDX_SA501X6XB00969	636	355	44.18%	PDX	Breast cancer	PDX	194.31
Laks2019	TNBC-PDX_SA535X5XB00517	928	444	52.16%	PDX	Breast cancer	PDX	447.63
Laks2019	TNBC-PDX_SA535X8XB01043	1,072	341	68.19%	PDX	Breast cancer	PDX	506.48
Laks2019	TNBC-PDX_SA604X6XB01979	968	476	50.83%	PDX	Breast cancer	PDX	487.41
Laks2019	TNBC-PDX_SA609X3XB01584	480	212	55.83%	PDX	Breast cancer	PDX	335.83
Laks2019	TNBC-PDX_SA609X4XB01721	606	392	35.31%	PDX	Breast cancer	PDX	388.63
Laks2019	TNBC-PDX_SA609X5XB01844	561	396	29.41%	PDX	Breast cancer	PDX	226.28
Laks2019	TNBC-PDX_SA609X6XB01898	626	410	34.50%	PDX	Breast cancer	PDX	188.30
Laks2019	TNBC-PDX_SA609X6XB01899	635	499	21.42%	PDX	Breast cancer	PDX	286.65
Laks2019	TNBC-PDX_SA609X7XB02184	844	634	24.88%	PDX	Breast cancer	PDX	772.79
Massey2022	GM12878	8,947	7,942	11.23%	GM12878	Lymphocyte	Cell line	288.45
Massey2022	GM12891	2,742	2,621	4.41%	GM12891	Lymphocyte	Cell line	170.15
Massey2022	GM12892	2,596	2,450	5.62%	GM12892	Lymphocyte	Cell line	165.07
Massey2022	H1	2,370	1,216	48.69%	H1	hESC	hESC	68.59
Massey2022	H7	1,923	1,780	7.44%	H7	hESC	hESC	234.20
Massey2022	H9	915	888	2.95%	H9	hESC	hESC	101.70
Massey2022	HCT-116	1,555	1,264	18.71%	HCT-116	Colon cancer	Cell line	73.84
Massey2022	MCF-7	1,337	982	26.55%	MCF-7	Breast cancer	Cell line	175.78
Massey2022	RKO	2,315	2,149	7.17%	RKO	Colon cancer	Cell line	124.94
Minussi2021	BT20	1,229	1,228	0.08%	BT20	Breast cancer	Cell line	215.55
Minussi2021	MDA-MB-157	1,210	1,210	0.00%	MDA-MB-157	Breast cancer	Cell line	243.43
Minussi2021	MDA-MB-231	2,710	2,710	0.00%	MDA-MB-231	Breast cancer	Cell line	252.10
Minussi2021	MDA-MB-453	1,260	1,260	0.00%	MDA-MB-453	Breast cancer	Cell line	235.28
Minussi2021	TN1	1,978	1,977	0.05%	Tumour	Breast cancer	Patient tumour	362.75
Minussi2021	TN2	1,024	1,023	0.10%	Tumour	Breast cancer	Patient tumour	542.82
Minussi2021	TN3	2,192	2,190	0.09%	Tumour	Breast cancer	Patient tumour	260.54
Minussi2021	TN4	1,301	1,301	0.00%	Tumour	Breast cancer	Patient tumour	225.59
Minussi2021	TN5	1,238	1,238	0.00%	Tumour	Breast cancer	Patient tumour	229.75
Minussi2021	TN6	1,205	1,205	0.00%	Tumour	Breast cancer	Patient tumour	218.43
Minussi2021	TN7	907	907	0.00%	Tumour	Breast cancer	Patient tumour	214.02
Minussi2021	TN8	1,224	1,224	0.00%	Tumour	Breast cancer	Patient tumour	222.53
Connolly2022	hTERT-RPE1	63	63	0.00%	hTERT-RPE1	Retinal pigment epithelial	Cell line	824.76
Takahashi2019	hTERT-RPE1	17	17	0.00%	hTERT-RPE1	Retinal pigment epithelial	Cell line	683.32
Funnell2022*	Pre-processed_CNV_hg19_SA039	878	878	0.00%	184-hTert	Mammary epithelial	Cell line	NA
Funnell2022*	Pre-processed_CNV_hg19_SA1054	382	382	0.00%	184-hTert	Mammary epithelial	Cell line	NA
Funnell2022*	Pre-processed_CNV_hg19_SA1055	391	391	0.00%	184-hTert	Mammary epithelial	Cell line	NA
Funnell2022*	Pre-processed_CNV_hg19_SA1056	496	496	0.00%	184-hTert	Mammary epithelial	Cell line	NA
Funnell2022*	Pre-processed_CNV_hg19_SA1188	2,003	2,003	0.00%	184-hTert	Mammary epithelial	Cell line	NA
Funnell2022*	Pre-processed_CNV_hg19_SA1292	404	404	0.00%	184-hTert	Mammary epithelial	Cell line	NA
Funnell2022*	Pre-processed_CNV_hg19_SA906a	3,711	3,711	0.00%	184-hTert	Mammary epithelial	Cell line	NA
Funnell2022*	Pre-processed_CNV_hg19_SA906b	5,716	5,716	0.00%	184-hTert	Mammary epithelial	Cell line	NA
Funnell2022*	Pre-processed_CNV_hg19_DG1134	133	133	0.00%	HGSC	HGSC	Patient tumour	NA
Funnell2022*	Pre-processed_CNV_hg19_DG1197	115	115	0.00%	HGSC	HGSC	Patient tumour	NA
Funnell2022*	Pre-processed_CNV_hg19_SA1049	1,283	1,283	0.00%	HGSC	HGSC	Patient tumour	NA
Funnell2022*	Pre-processed_CNV_hg19_SA1091	506	506	0.00%	HGSC	HGSC	Patient tumour	NA
Funnell2022*	Pre-processed_CNV_hg19_SA1096	802	802	0.00%	HGSC	HGSC	Patient tumour	NA
Funnell2022*	Pre-processed_CNV_hg19_SA1162	254	254	0.00%	HGSC	HGSC	Patient tumour	NA
Funnell2022*	Pre-processed_CNV_hg19_SA1180	774	774	0.00%	HGSC	HGSC	Patient tumour	NA
Funnell2022*	Pre-processed_CNV_hg19_SA1182	214	214	0.00%	HGSC	HGSC	Patient tumour	NA
Funnell2022*	Pre-processed_CNV_hg19_SA1050	990	990	0.00%	HGSC	HGSC	Patient tumour	NA
Funnell2022*	Pre-processed_CNV_hg19_SA1051	892	892	0.00%	HGSC	HGSC	Patient tumour	NA
Funnell2022*	Pre-processed_CNV_hg19_SA1052	556	556	0.00%	HGSC	HGSC	Patient tumour	NA
Funnell2022*	Pre-processed_CNV_hg19_SA1053	825	825	0.00%	HGSC	HGSC	Patient tumour	NA
Funnell2022*	Pre-processed_CNV_hg19_SA1181	296	296	0.00%	HGSC	HGSC	Patient tumour	NA
Funnell2022*	Pre-processed_CNV_hg19_SA1184	621	621	0.00%	HGSC	HGSC	Patient tumour	NA
Funnell2022*	Pre-processed_CNV_hg19_SA1047	347	347	0.00%	HGSC	HGSC	Patient tumour	NA
Funnell2022*	Pre-processed_CNV_hg19_SA1093	346	346	0.00%	HGSC	HGSC	Patient tumour	NA
Funnell2022*	Pre-processed_CNV_hg19_SA530	324	324	0.00%	TNBC	Breast cancer	Patient tumour	NA
Funnell2022*	Pre-processed_CNV_hg19_SA604	2,139	2,139	0.00%	TNBC	Breast cancer	Patient tumour	NA
Funnell2022*	Pre-processed_CNV_hg19_SA609	6,033	6,033	0.00%	TNBC	Breast cancer	Patient tumour	NA
Funnell2022*	Pre-processed_CNV_hg19_SA610	268	268	0.00%	TNBC	Breast cancer	Patient tumour	NA
Funnell2022*	Pre-processed_CNV_hg19_SA501	2,473	2,473	0.00%	TNBC	Breast cancer	Patient tumour	NA
Funnell2022*	Pre-processed_CNV_hg19_SA535	1,801	1,801	0.00%	TNBC	Breast cancer	Patient tumour	NA
Funnell2022*	Pre-processed_CNV_hg19_SA605	65	65	0.00%	TNBC	Breast cancer	Patient tumour	NA
TOTAL		134,168	119,991					

*Duplicates from Laks2019 not included.
