# Supplementary material for: Unravelling single-cell DNA replication timing dynamics using machine learning reveals heterogeneity in cancer progression
Source: Nat Commun. 2025 Feb 8;16:1472. doi: 10.1038/s41467-025-56783-0 (PMC11807193; doi:10.1038/s41467-025-56783-0)
Supplement: Supplementary file 2 — Reporting Summary [file 41467_2025_56783_MOESM2_ESM.pdf]

nature portfolio

Corresponding author(s): Chun-Long CHEN

Last updated by author(s): Nov 28, 2024

Reporting Summary

Nature Portfolio wishes to improve the reproducibility of the work that we publish. This form provides structure for consistency and transparency in reporting. For further information on Nature Portfolio policies, see our Editorial Policies and the Editorial Policy Checklist.

Statistics

For all statistical analyses, confirm that the following items are present in the figure legend, table legend, main text, or Methods section.

n/a	Confirmed	
☐	☒	The exact sample size (*n*) for each experimental group/condition, given as a discrete number and unit of measurement
☐	☒	A statement on whether measurements were taken from distinct samples or whether the same sample was measured repeatedly
☐	☒	The statistical test(s) used AND whether they are one- or two-sided<br>*Only common tests should be described solely by name; describe more complex techniques in the Methods section.*
☒	☐	A description of all covariates tested
☒	☐	A description of any assumptions or corrections, such as tests of normality and adjustment for multiple comparisons
☐	☒	A full description of the statistical parameters including central tendency (e.g. means) or other basic estimates (e.g. regression coefficient) AND variation (e.g. standard deviation) or associated estimates of uncertainty (e.g. confidence intervals)
☐	☒	For null hypothesis testing, the test statistic (e.g. F, t, r) with confidence intervals, effect sizes, degrees of freedom and P value noted<br>*Give P values as exact values whenever suitable.*
☒	☐	For Bayesian analysis, information on the choice of priors and Markov chain Monte Carlo settings
☒	☐	For hierarchical and complex designs, identification of the appropriate level for tests and full reporting of outcomes
☐	☒	Estimates of effect sizes (e.g. Cohen's *d*, Pearson's *r*), indicating how they were calculated

Our web collection on statistics for biologists contains articles on many of the points above.

Software and code

Policy information about availability of computer code

Data collection	See Data Availability section, methods and Supplementary Table 3.
Data analysis	Software versions appear in the Methods section. Code used for the analysis is provided in the Code availability section on Github.<br>List of software used:<br>samtools 1.16.1<br>demultiplex 1.2.2<br>R 4.0.4 and 4.3.3<br>cutoff 0.1.0<br>Trim Galore 0.6.4<br>Cutadapt 3.7<br>FastQC 0.11.9<br>BWA 0.7.17<br>Picard 2.26.11<br>java 19<br>MultiQC 1.10.1<br>Keras 2.13.1<br>Python 3.9.11 and 3.11.8<br>PHATE 1.0.7<br>scipy 1.12.0<br>MnM 1.0.0

For manuscripts utilizing custom algorithms or software that are central to the research but not yet described in published literature, software must be made available to editors and reviewers. We strongly encourage code deposition in a community repository (e.g. GitHub). See the Nature Portfolio guidelines for submitting code & software for further information.

Data

Policy information about availability of data

All manuscripts must include a data availability statement. This statement should provide the following information, where applicable:

- Accession codes, unique identifiers, or web links for publicly available datasets
- A description of any restrictions on data availability
- For clinical datasets or third party data, please ensure that the statement adheres to our policy

Our data availability section contains the following text:
The 10x barcode whitelist can be found at https://github.com/TheKorenLab/Single-cell-replication-timing/blob/main/align/10x_barcode_whitelist.txt, the human reference genome hg38 at https://support.illumina.com/sequencing/sequencing_software/igenome.html, and the genomic blacklist at https://github.com/Boyle-Lab/Blacklist. Published scWGS datasets can be found in the Gene Expression Omnibus (GEO) under the accession numbers GSE186173 [https://www.ncbi.nlm.nih.gov/geo/query/acc.cgi?acc=GSE186173]37, GSE158011 [https://www.ncbi.nlm.nih.gov/geo/query/acc.cgi?acc=GSE158011]32, GSE108556 [https://www.ncbi.nlm.nih.gov/geo/query/acc.cgi?acc=GSE108556]35 and in the Sequence Read Archive (SRA) under PRJNA770772 [https://www.ncbi.nlm.nih.gov/sra/?term=PRJNA770772]38. Access to BC Cancer datasets is controlled and requires a data access agreement which can be found at the European Genome-Phenome archive (EGA) under the accession number EGAS00001003190 [https://ega-archive.org/studies/EGAS00001003190]22. Processed scCNV data from ref.26 can be found at https://zenodo.org/record/6998936. Bulk RT profiles were obtained under accession numbers GSE34399 [https://www.ncbi.nlm.nih.gov/geo/query/acc.cgi?acc=GSE34399] for MCF-7 and GSE158011 [https://www.ncbi.nlm.nih.gov/geo/query/acc.cgi?acc=GSE158011] for HCT-116 cells. The liftover chain file is available at https://hgdownload.cse.ucsc.edu/goldenpath/hg19/liftOver. Source data are provided with this paper. The scRT/scCNV atlas generated in this study can be found on MnM's GitHub page [https://github.com/CL-CHEN-Lab/MnM/tree/main/scRT_scCNV_Atlas].

Research involving human participants, their data, or biological material

Policy information about studies with human participants or human data. See also policy information about sex, gender (identity/presentation), and sexual orientation and race, ethnicity and racism.

Reporting on sex and gender	Copy-number data on chromosomes X and/or Y could be used to determine sex. Data was collected on availability of published data and not selected based on other criteria. Generation of Replication Timing profiles did not consider sex chromosomes because only autosomal data was comparable.
Reporting on race, ethnicity, or other socially relevant groupings	Unknown: Patient information was not disclosed and not considered in this study.
Population characteristics	Unknown: Patient information was not disclosed.
Recruitment	Data was collected based on availability of published data and not selected based on other criteria. No individuals were recruited in this study.
Ethics oversight	Part of this manuscript was prepared using a limited access dataset obtained from BC Cancer and does not necessarily reflect the opinions or views of BC Cancer.

Note that full information on the approval of the study protocol must also be provided in the manuscript.

Field-specific reporting

Please select the one below that is the best fit for your research. If you are not sure, read the appropriate sections before making your selection.

☒ Life sciences ☐ Behavioural & social sciences ☐ Ecological, evolutionary & environmental sciences

For a reference copy of the document with all sections, see nature.com/documents/nr-reporting-summary-flat.pdf

Life sciences study design

All studies must disclose on these points even when the disclosure is negative.

Sample size	The study analyzed over 119,000 single cells sourced from a variety of human-derived samples, including cultured cell lines, patient tumors, and patient-derived xenografts. This sample size was selected to ensure a robust and diverse representation of single-cell DNA replication timing (scRT) dynamics across multiple cancer types and subpopulations. Data was collected based on availability of datasets and sample sizes are reported in Supplementary Table 3.
Data exclusions	Data exclusions (quality-control) are listed in the Methods section and Supplementary Table 3.

Replication	Key aspects of the study, including missing-value imputation, cell replication state classification, and subpopulation clustering, were validated individually to confirm the accuracy and consistency. The use of random seeds was used for replication of the results (see Methods).
Randomization	Randomization was not applicable in this study, as the study focused on analyzing pre-existing, heterogeneous samples (cancer cell lines, patient tumors, and PDX samples) without introducing any experimental treatment or control groups. For the deep learning model, data was randomly split into training and test sets (see Methods).
Blinding	Blinding was not performed, as the study was based on computational analysis of single-cell genomic data and did not involve subjective assessments. Analyses were conducted in an unbiased manner using predefined machine learning algorithms that automated classification.

Reporting for specific materials, systems and methods

We require information from authors about some types of materials, experimental systems and methods used in many studies. Here, indicate whether each material, system or method listed is relevant to your study. If you are not sure if a list item applies to your research, read the appropriate section before selecting a response.

Materials & experimental systems

n/a	Involved in the study	
☒	☐	Antibodies
☐	☒	Eukaryotic cell lines
☒	☐	Palaeontology and archaeology
☐	☒	Animals and other organisms
☒	☐	Clinical data
☒	☐	Dual use research of concern
☒	☐	Plants

Methods

n/a	Involved in the study	
☒	☐	ChIP-seq
☒	☐	Flow cytometry
☒	☐	MRI-based neuroimaging

Eukaryotic cell lines

Policy information about cell lines and Sex and Gender in Research

Cell line source(s)	Data from cell lines were obtained from published and public datasets. No cell-lines were used in this computational study.
Authentication	N/A: No cell-lines were used in this computational study.
Mycoplasma contamination	N/A: No cell-lines were used in this computational study.
Commonly misidentified lines (See ICLAC register)	N/A: No cell-lines were used in this computational study.

Animals and other research organisms

Policy information about studies involving animals; ARRIVE guidelines recommended for reporting animal research, and Sex and Gender in Research

Laboratory animals	No animals were used in this computational study.
Wild animals	No animals were used in this computational study.None
Reporting on sex	N/A: Replication timing generation excluded sex chromosomes in this study.
Field-collected samples	None
Ethics oversight	None. No animals were used in this computational study.

Note that full information on the approval of the study protocol must also be provided in the manuscript.

Plants

Seed stocks	None
Novel plant genotypes	None
Authentication	None
