# Supplementary material for: Unravelling single-cell DNA replication timing dynamics using machine learning reveals heterogeneity in cancer progression
Source: Nat Commun. 2025 Feb 8;16:1472. doi: 10.1038/s41467-025-56783-0 (PMC11807193; doi:10.1038/s41467-025-56783-0)
Supplement: Supplementary file 3 — Machine Learning Reporting Summary [file 41467_2025_56783_MOESM3_ESM.pdf]

Corresponding author(s): Chun-Long CHEN
Last updated by author(s): 31/10/2024

# Machine Learning Reporting Summary

## 1. Availability and reproducibility of Code and Data

Please select all that apply regarding the availability of the data and code used in the study.

- ☐ Code will be included in a CodeOcean capsule.
- 🗹 The source code is included in the submission or available in a public repository: https://github.com/CL-CHE (url)
- 🗹 A compiled standalone version of the software is included in the submission or available in a public repository: https://github.com/CL-CHEN-Lab/M (url)
- 🗹 A test dataset and instructions/scripts for replicating the results are included in the submission or available in a public repository: https://github.com/CL-CHEN-Lab/Mr (url)
- 🗹 A Readme file with instructions for installing and running the code is included in the submission or available in a public repository: https://github.com/CL-CHEN-Lab/Mr (url)
- 🗹 The code is made available to reviewers during review.
- 🗹 Pretrained models are used in the study and accessible through: https://github.com/ (url)
- ☐ Pretrained models are used in the study and are not accessible.
- 🗹 The paper contains information on how to obtain code and data after publication.

## 2. Datasets

A. All data sources are listed in the paper.
   - 🗹 Yes
   - ☐ No (please justify) ____________________

B. The train, test and validation datasets are publicly available and links/accession numbers have been provided in the manuscript or supplementary materials.
   - 🗹 Yes
   - ☐ No (please justify) ____________________

C. We have reported and discussed potential dataset biases in the paper. Where applicable, appropriate mitigation strategies were used.
   - 🗹 Yes (please specify section) ____________________
   - ☐ No (please justify) ____________________

D. The data cleaning and preprocessing steps are clearly and fully described, either in text or as a code pipeline.

☑ Yes (please specify section) Methods and Code

☐ No (please justify) ______

E. Instances of combining data from multiple sources are clearly identified, and potential issues mitigated.

☑ Yes (please specify section) Discussion

☐ No (please justify) ______

## 3. Model and training

A. What model architecture is the current model based on? Sequential Model (FNN), DBSC

B. A Model Card is provided[1].

☐ Yes

☑ No

C. The model clearly splits data into different sets for training (model selection), validation (hyperparameter optimization), and testing (final evaluation).

☑ Yes

☐ No

D. The method of data splitting (e.g. random, cluster- or time-based splitting, forward cross-validation) is clearly stated.

☑ Yes (please specify) Available in the Code: test/training split randomly

☐ No (please justify) ______

E. The data splitting mimics anticipated real-world applications.

☑ Yes

☐ No

F. The data splitting procedure has been chosen to avoid data leakage.

☑ Yes (please specify) Test and training sets do not contain the same data (Methoc

☐ No (please justify) ______

G. The interpretability of the model has been studied and clearly validated.

☑ Yes (please specify section) Results

☐ No (please justify) ______

## 4. Evaluation

A. The performance metrics used are described and justified in the paper.

☑ Yes (please specify section) Results and Methods

☐ No (please justify) ______

[1] https://huggingface.co/docs/hub/model-cards

B. Cross-validation of the results is included.
 ☑ Yes
 ☐ No
C. Community-accepted benchmark datasets/tasks are used for comparisons.
 ☑ Yes (please specify) Accuracy compared to results from Massey & Koren NC 202
 ☐ No
D. Baseline comparisons to simple/trivial models (for example, 1-nearest neighbour, random forest, most frequent class) are provided.
 ☑ Yes
 ☐ No
E. Benchmarks with current state-of-the-art are provided.
 ☑ Yes
 ☐ No
F. Ablation experiments are included.
 ☐ Yes
 ☑ No
G. The model has been tested on a fully independent dataset.
 ☑ Yes
 ☐ No

## 5. Computational resources

A. The paper contains information on hardware/computing resources that were used.
 ☑ Yes
 ☐ No
B. The paper includes information on the computational costs in terms of computation time, parallelization or carbon footprints estimates.
 ☑ Yes
 ☐ No
